# Sublethal Effects of Imidacloprid on Honey Bee Colony Growth and Activity at Three Sites in the U.S.

**DOI:** 10.1371/journal.pone.0168603

**Published:** 2016-12-28

**Authors:** William G. Meikle, John J. Adamczyk, Milagra Weiss, Ales Gregorc, Don R. Johnson, Scott D. Stewart, Jon Zawislak, Mark J. Carroll, Gus M. Lorenz

**Affiliations:** 1 Carl Hayden Bee Research Center, USDA-ARS, Tucson, AZ United States of America; 2 Southern Horticultural Laboratory, USDA-ARS, Poplarville, MS United States of America; 3 Mississippi State University, South MS Branch Experiment Station, Poplarville, MS United States of America; 4 University of Arkansas Division of Agriculture Cooperative Extension Service, Lonoke Res. & Ext. Ctr., Lonoke, AR United States of America; 5 The University of Tennessee, West Tennessee Research & Education Center, 605 Airways Blvd, Jackson, TN United States of America; 6 University of Arkansas Division of Agriculture Cooperative Extension Service, 2301 South University, Little Rock, AR United States of America; Montana State University Bozeman, UNITED STATES

## Abstract

Imidacloprid is a neonicotinoid pesticide heavily used by the agricultural industry and shown to have negative impacts on honey bees above certain concentrations. We evaluated the effects of different imidacloprid concentrations in sugar syrup using cage and field studies, and across different environments. Honey bee colonies fed sublethal concentrations of imidicloprid (0, 5, 20 and 100 ppb) over 6 weeks in field trials at a desert site (Arizona), a site near intensive agriculture (Arkansas) and a site with little nearby agriculture but abundant natural forage (Mississippi) were monitored with respect to colony metrics, such as adult bee and brood population sizes, as well as pesticide residues. Hive weight and internal hive temperature were monitored continuously over two trials in Arizona. Colonies fed 100 ppb imidacloprid in Arizona had significantly lower adult bee populations, brood surface areas and average frame weights, and reduced temperature control, compared to colonies in one or more of the other treatment groups, and consumption rates of those colonies were lower compared to other colonies in Arizona and Arkansas, although no differences in capped brood or average frame weight were observed among treatments in Arkansas. At the Mississippi site, also rich in alternative forage, colonies fed 5 ppb imidacloprid had less capped brood than control colonies, but contamination of control colonies was detected. In contrast, significantly higher daily hive weight variability among colonies fed 5 ppb imidacloprid in Arizona suggested greater foraging activity during a nectar flow post treatment, than any other treatment group. Imidacloprid concentrations in stored honey corresponded well with the respective syrup concentrations fed to the colonies and remained stable within the hive for at least 7 months after the end of treatment.

## Introduction

Beekeepers and the public have raised concerns about the effects of neonicotinoids, a class of neurotoxic insecticides used as systemic pesticides, on honey bee health. The use of neonicotinoids in agricultural and urban environments has grown dramatically since the introduction of the first, imidacloprid, in 1994; at present, neonicotinoids account for more than 15% of global pesticide sales [1, 2] and imidacloprid is now a top-selling pesticide globally [3]. Neonicotinoids are applied as field sprays and seed treatments [4], so honey bees may be exposed via direct application, contact with treated surfaces, seed dusts and plant products including pollen, nectar and exudate from extra-floral nectaries [3–8]. Acute toxicity is clearly detrimental to honey bees, but the LC_50_ of imidacloprid, 1760 ng/L, is above what is considered a “field realistic” range [9]. However, sublethal effects of neonicotinoids have been observed in laboratory experiments [1] and field studies [9, 10], although few studies have examined colony-level behaviors such as foraging activity or temperature control. Sublethal neonicotinoid pesticides can directly impair honey bee learning and sensory capabilities [11, 12], decrease foraging success and survivorship [8, 10], and can have indirect effects on bees by acting as repellents [13]. Dively et al. [2] reported effects of sublethal imidacloprid concentrations in pollen diet, which is mainly consumed by newly-emerged bees and nurse bees on brood production, queen replacement, foraging activity and winter survivorship. Pesticides can interact with pests and pathogens; bees exposed to neonicotinoids have been found to have higher *Varroa* and *Nosema* densities [2, 14–16]. Synergistic effects of imidacloprid with a pyrethroid on pollinators has also been observed [17]. While size of the worker bee population is often the main criterion used by beekeepers for judging colony health, sublethal pesticide exposure may affect many aspects of honey bee ecology and social organization such as temperature regulation [18]. Sublethal pesticide exposure may have delayed effects as the colony exploits nutritional reserves in honey, pollen, and the bodies of the workers themselves. Few colony-level effects of field-relevant concentrations of imidacloprid exposure via sugar syrup, which is consumed throughout the life of the adult bee [19], have been reported. Identifying symptoms of sublethal exposure on colony-level behavior would help beekeepers detect such exposure.

Two bee cage studies were conducted to measure survivorship and food consumption by newly-emerged adult bees exposed to various concentrations of imidacloprid in sugar syrup. Four field studies were conducted in which honey bee colonies were exposed to imidacloprid concentrations in three different landscapes in the southern half of the U.S. for a qualitative examination of the role of environment. Two studies were conducted at sites in Arkansas and Mississippi with abundant forage, both agricultural and natural, and two studies conducted at a relatively isolated site in southern Arizona with no commercial agriculture and little natural forage during the treatment period. Capped brood levels and other metrics of colony phenology and size were measured at all sites, and continuous weight and temperature data, which have been shown to reflect honey bee colony growth, adult bee population size, foraging activity and brood production [20, 21] were monitored at the Arizona sites. Coumaphos, often used against bee pests and a common contaminant of hive products [22, 23], was applied to colonies in Mississippi before the imidacloprid application and those data were evaluated for interactions with imidacloprid. Proper apiary management at each site was given primacy over consistency across sites. Imidacloprid concentrations were chosen to include both a low (5 ppb) concentration, observed in field samples [1] and a concentration high enough (100 ppb) was used here to increase the chances of provoking a response in terms of hive growth and activity, and to harmonize to some extent this study with the experimental designs of other workers (e.g., [2]). A summary of the experiments is provided (S1 Table).

## Materials and Methods

### Cage studies

Two cage studies were conducted: the first from 11 Sept. to 31 Oct. 2014 in which groups of 100 bees were fed sugar syrup (see S2 Table) containing either 100, 5 or 0 ppb imidacloprid (10 replicate cages per treatment) and the second from 27 July to 16 Sept 2015 in which groups were fed syrup containing either 100, 20, 5 or 0 ppb imidacloprid (7–8 replicate cages per treatment). In each experiment, one frame of mature brood was removed from each of four 6-month-old colonies with Cordovan Italian queens (C.F. Koehnen & Sons, Inc) established at the Carl Hayden Bee Research Center, Tucson, AZ and placed in an incubator (Percival model I36VL) at 30°C and 50% r.h. Adult bees emerging over the following 48 h were distributed among 30 Plexiglas® cages (internal volume 785 cm^3^) until each cage had 100 bees. Each cage had plastic feeding bottles on top containing 15 mL sugar solution and 50 mL water, and a 4x4 cm square of wax foundation hung vertically in the center. A mixture of 10g each of pollen (Natural Foods Inc., Toledo, OH), sucrose and inverted sucrose was placed inside a rubber gasket accessed via a hole in the side of the cage. Dead bees were removed and counted daily. Syrup consumption was measured weekly by weighing bottles of syrup before and after use; vials were emptied and refilled with fresh syrup. Consumption per bee was calculated as the observed consumption for a given cage divided by the number of “bee-days” for that time period, in which a bee-day represents one bee alive for one day in that cage. Water consumption was measured the same way but less often and only total water consumption was analyzed. Five to eight live bees were removed from each cage after 4 and 6 weeks; those data were censored. Bee samples were submitted to the Laboratory Approval and Testing Division, Agricultural Marketing Service, USDA (LATD) for residue analysis. Adult bee survivorship in cage studies was analyzed using Proc LifeReg (SAS Inc. 2002). An appropriate distribution was first chosen to model survivorship, survivorship curves were generated for each replicate cage based on that model, and treatments compared using ANOVA (Proc Glimmix, SAS Inc. 2002) with respect to the 30^th^ and 50^th^ percentiles, and shape was estimated by subtracting the 40^th^ percentile from the 30^th^ percentile. Weekly syrup consumption per bee was analyzed using repeated measures MANOVA.

### Field experiments—Arizona

Two studies were conducted. In the first study, from May 2014 to March 2015 (hereafter 2014 experiment), bee colonies were fed sugar syrup containing either 100, 5 or 0 ppb imidacloprid. In the second study, from June to September 2015 (hereafter the 2015 experiment), colonies were fed syrup containing either 100, 20, 5 or 0 ppb imidacloprid. In each study, N = 4 bee colonies per treatment group. All colonies were established from packages (C.F. Koehnen & Sons, Inc., Glenn, CA 95943) three months prior to treatment in painted, 10-frame, wooden Langstroth boxes (43.7 l capacity) (Mann Lake Ltd,) with migratory wooden lids at the Santa Rita Experimental Range south of Tucson, AZ (31°46'39"N, 110°51'46"W). At establishment each colony was given three frames of drawn comb with six frames of plastic comb foundation and fed 2 kg sugar syrup (1:1 w:w) and 250 g pollen patty (see Supplementary material 1). The apiary was surrounded by native, unmanaged plants, particularly mesquite (*Prosopis* spp.). After 4–6 weeks, hives were placed on stainless steel electronic scales (TEKFA® model B-2418 and Avery Weigh-Tronix model BSAO1824-200) (max. capacity 100 kg) connected to 12-bit dataloggers (Hobo® U-12, Onset Computer Corporation), set to record weight every 15 minutes. The system had an overall precision of approximately ±20 g. On the same day, a temperature sensor (iButton Thermochron, precision ±0.06°C) enclosed in brass mesh was stapled to the center of the top bar on the 5th frame in each hive and set to record every 30 min. Hives were inspected at 4-week intervals starting 5 weeks before treatment, using a published protocol [21]. For each inspection, frames were gently shaken to dislodge adult bees, then weighed, photographed using a 16.3 megapixel digital camera (Pentax K-01, Ricoh Imaging Co., Ltd.) and replaced in the hive. The area of sealed brood per frame was estimated from the photographs using ImageJ version 1.47 software (W. Rasband, National Institutes of Health, USA). The total weight of the adult bee population was calculated by subtracting the combined weights of hive components (i.e. lid, inner cover, box, bottom board, frames, entrance reducer, internal feeder) obtained at the start of the experiment (model EC15, OHaus) from the total hive weight recorded the midnight prior to the inspection. At each inspection, 3–5 g of adult bees, wax and honey were each collected from each hive into 50 ml centrifuge tubes and stored at -20°C. Wax was collected from all frames with comb pre-treatment, and from recently built comb during and post treatment. Adult bees were collected from a frame next to the brood cluster, and nectar was collected from capped cells pre-treatment and from recently filled cells during and post-treatment. Pooled samples collected prior to treatment were analyzed for residues of 174 compounds by LATD; later samples were analyzed only for neonicotinoid pesticides and breakdown products.

Hives were separated into four groups and randomly assigned to treatment group. Hives within a group were 0.5–1 m apart and groups were >3 m apart. Just prior to treatment all broodless frames containing honey and/or pollen were replaced with frames of empty drawn comb collected earlier from the same apiary, leaving colonies with an average (±s.e.) of 1620 (±246) g food in 2014 and 1561 (±145) g food in 2015. Disposable latex gloves, hive tools and sampling equipment used in hive inspections were changed for each treatment group; hives were inspected from lowest to highest concentration treatments. Starting 17 July 2014 for the first experiment and 9 July 2015 for the second, colonies were given 2 kg of treated syrup twice per week for the first 2 weeks and 3 kg twice per week for the last 4 weeks, and not fed thereafter. Infestation levels of *Varroa destructor* were monitored on four occasions during and post treatment in 2014 and once during treatment in 2015 by placing adhesive cardboard strips (Mann Lake Ltd) under the brood box for 3 d and counting the mites. Colonies in the first experiment were treated with amitraz (Apivar®) on 4 Nov. The 2015 study was terminated shortly after the end of treatment owing to visits by a large animal, probably a bear.

Continuous data were divided into daily average data and within-day detrended data. Detrended data were calculated as the difference between the 25 hour running average and the raw data [21]. Sine curves were fit 3-day subsamples of detrended data taken sequentially by day (see [21]) and curve amplitudes, representing estimates of daily variability, were used as a response variables. Weight and temperature amplitude datasets were reduced to a data point every 5 days for repeated measures analysis to ensure no overlap between 3 d samples. For consistency, running average data were treated in the same fashion. Ambient weather data for 2014 was obtained for comparison (AmeriFlux US-SRM Santa Rita Mesquite, doi:10.17190/AMF/1246104).

### Field experiment-Arkansas

In May 2015, 16 honey bee colonies were established from packages with Italian queens from the same breeding line obtained from a commercial breeder (Diamond Lakes Apiaries, Murfreesboro, AR) in new 10-frame Langstroth deep boxes (Dadant & Sons, Inc., IL) with plastic comb foundation near Lonoke, AR (34°40'38"N, 91°55'21"W) and fed sugar syrup (1:2 w:w). Forage consisted of cotton, soybeans and rice in addition to trees and wildflowers. Colonies were inspected prior to treatment on 7 July. At each inspection, adult bee, honey and wax were sample, and hive frames weighed and photographed, as described above for the Arizona experiments; samples were analyzed for all pesticide residues at LATD. Honey frames were not removed prior to treatment. Amitraz (Apivar) strips were installed in colonies prior to treatment, and the strips left in place during syrup feeding. Hives were weighed 14 July and every 14–18 days thereafter until 29 Sept. Colonies were randomly assigned to treatment groups and starting 15 July were fed 2 kg sugar syrup with imidacloprid concentrations of 0, 5, 20 or 100 ppb twice per week, until 3 Aug., after which the quantity of syrup was increased to 3 kg twice per week until 24 Aug. Hives were inspected on 6 Aug., 28–29 Aug. and 29–30 Sept, and samples taken on 31 Aug. and 23 Sept.

### Field experiment-Mississippi

In April 2015, 15 honey bee colonies, (Group A) were established from packages with Italian queens from the same breeding line (Gunter Honey, Inc., Lumberton, MS) in new 10-frame Langstroth deep boxes (Dadant & Sons, Inc.) with plastic comb foundations at the Mississippi State University Coastal Experiment Station in McNeill, MS (30°39'46"N, 89°38'01"W). Forage consisted of Chinese tallow trees (*Triadica sebifera*) in June and goldenrod (*Solidago* sp.) in Oct. In June a second group of 13 bee colonies (Group B) was established at the same site. Group A colonies were inspected 21 May and 23 June. At each inspection, hive strength was estimated by counting “frame spaces” (spaces between frames occupied by adult bees, with the spaces between the frame and box on each side being 0.5 spaces each; the values are whole numbers between 0 and 10 inclusive). Capped brood surface area was measured by placing a transparent plastic sheet marked with regular hexagons (23.4 cm^2^ each) on the comb and counting occupied hexagons, rounding up if more than half filled (see [24]). Adult bees were sampled near brood frames and honey was sampled from brood frames; samples were stored at -20°C. *Varroa* densities were assessed in Aug. and Oct. by collecting ≥230 adult bees into 1 L Mason jars with 70% ethanol, agitating the jar, and counting mites and bees.

Eight randomly-selected colonies from Group A received 1 kg carbohydrate patty (Pro Winter, Mann Lake Ltd) mixed with coumaphos at 5.8 ppm (see S2 Table), a concentration reported from pollen [23], on 21 May, and again four weeks later. The remaining seven colonies received coumaphos-free carbohydrate patties. On 16 July, all 28 Group A and Group B colonies were inspected and divided into seven treatment groups of four colonies each. Frames containing only stored honey were replaced with empty drawn comb. On 17 July, the eight Group A colonies exposed to coumaphos were split into two groups, with four colonies getting coumaphos at 5.8 ppm in addition to imidacloprid at 5 ppb, and the other four getting 5.8 ppm coumaphos plus 20 ppb imidacloprid. Of the seven colonies in Group A that did not get coumaphos, four were given 5.8 ppm coumaphos syrup and the remaining three colonies given syrup with no additives. The same day, the 13 colonies in Group B were divided into three groups of four colonies, and the colonies received syrup with imidacloprid at 5, 20 or 100 ppb, respectively, with the remaining colony included as an untreated control (see S1 Fig). During treatment, each colony received 1 kg syrup three times per week until the end of treatment period on 19 Aug. for a cumulative total of 12 kg. All 28 colonies were inspected on 17 Aug.,17 Sept. and 19 Oct. Colonies received a further five 1 kg feedings of 2:1 sugar syrup from 20 Aug. to 14 Oct. Adult bee and honey samples were pooled within treatment group for each sampling date and analyzed at LATD for residues of imidacloprid, coumaphos and breakdown products. The effects of treatment on brood surface area were evaluated using mixed-model ANOVA, with frame space estimates prior to treatment used as covariates. Frame spaces were analyzed using non-parametric tests (Proc Npar1way, SAS Inc. 2002).

#### Study sites

Use of the apiary site in Arizona was granted through Range Use Agreement with the University of Arizona Agricultural Experiment Station, Santa Rita Experimental Range. Use of the apiary site in Arkansas was granted through an agreement with a private landowner and the site is registered with the Arkansas State Plant Board, in accordance with Arkansas apiary law. The use of the apiary site in Mississippi was granted through the USDA Agricultural Research Service. The land use did not involve endangered or protected species.

## Results

### Cage studies

Experiments were terminated after 50 d with 25–70 remaining alive per cage in 2014 and 23–64 per cage in 2015. Bees collected before treatment in both trials tested negative for neonicotinoid pesticides or breakdown products, and no imidacloprid was detected in any samples from 2015. Only bees collected from the 100 ppb group in 2014 during treatment tested positive (< 2 ppb), possibly due to contaminated syrup in their digestive tracts. A Weibull distribution with censoring was selected to model survivorship curves for each replicate. The intercept and scale parameters from the fit for each replicate were significant (P<0.001). The standard error of the intercept as a percentage of the intercept ranged from 1.1 to 10.9% for the 2014 data and from 1.1 to 6.8% for the 2015 data. For the scale parameter those values ranged from 5.2 to 24.9% for the 2014 data and from 10.7 to 20.7% for the 2015 data (see Figs A-D in S1 File). The 0, 5 and 100 ppb treatments were analyzed across both experiments, and the analysis was also conducted using the 0, 5, 20 and 100 ppb data from the second experiment. Treatment did not have a significant effect on the 30^th^ percentile, 50^th^ percentile, or shape response variables for either analysis (S3 Table).

**Fig 1:**
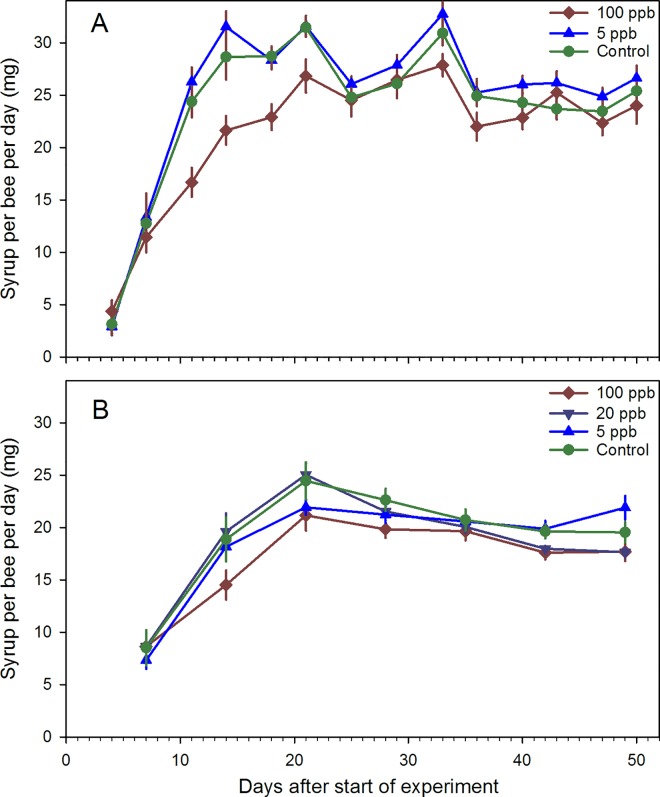
Average daily consumption per day per honey bees. Adult honey bees were kept in cages and fed sugar syrup with imidacloprid concentrations of either 100 ppb, 20 ppb, 5 ppb or 0 ppb. Each cage was stocked with 100 bees (7–8 cages per group). A) Experiment conducted Sept-Oct. 2014 (no 20 ppb treatment); B) Experiment conducted Aug.-Sept. 2015.

Syrup and water consumption were likewise compared 1) across experiments for the 0, 5 and 100 ppb groups; and 2) within the second experiment for the 0, 5, 20 and 100 ppb groups. In the first analysis, treatment (F_2,347_ = 19.68, P<0.0001) and experiment (F_1,349_ = 182.40, P<0.0001) were significant (Fig 1). Bees in the 100 ppb group consumed less syrup on average than those in either the 5 ppb or control groups (P<0.0001 for both). In the second analysis, no significant differences were observed (P = 0.14). Water consumption was significantly different among groups in the first analysis (F_2,47_ = 3.79, P = 0.0298) and between years (F_1,47_ = 36.07, P<0.0001) (S2 Fig). Bees in the 100 ppb group drank more water than bees in the control (P = 0.0419) and bees in 2015 drank more than those in 2014. In the second analysis, water consumption was significantly different among groups (F_3,26_ = 3.20, P = 0.0400) but no pairwise contrasts were significant.

### Field experiments–Arizona

No agrochemical residues were detected in adult bees or honey in 2014, but samples of wax pooled among hives contained miticides fluvalinate (32–34 ppb) and coumaphos (9 ppb) and the fungicide carbendazim (16–65 ppb). One sample also had an insect growth regulator (IGR) hydroprene, at 144 ppb. Wax samples from the 2015 experiment contained four varroacides: fluvalinate (up to 781 ppb), thymol (up to 648 ppb), fenpyroximate (up to 33 ppb) and trace amounts of coumaphos, as well as carbendazim (up to 27 ppb) and trace amounts of hexythiazox, an IGR. Honey from 2015 showed trace carbendazim. Thymol was detected in adult bees (194–392 ppb) but not applied. Imidacloprid was detected in adult bees only in the 100 ppb treatment in both field experiments (Table 1). In 2014, imidacloprid concentrations remained largely stable in stored honey through the following March. Imidacloprid was detected in low levels in wax in the100-ppb group post treatment in 2014 but not in 2015.

**Table 1:** Imidacloprid concentrations, in ppb, in honey, adult bees and wax before (July), during (Aug.) and after exposure of honey bee colonies to contaminated syrup in Arizona.

Year	Material	Conc.	17 July	13 Aug.	9 Sept.	7 Oct.	4 Nov.	26 Feb.
2014	Honey	100 ppb	-	33.1	130.0	76.9	99.7	104.0
		5 ppb	-	-	5.8	3.3	6.1	5.9
		Control	-	-	-	-	-	4.2
	Adult bees	100 ppb	-	-	19.3	10.8	13.6	6.8
		5 ppb	-	-	-	-	-	-
		Control	-	-	-	-	-	-
	Wax	100 ppb	-	9.0	3.9	5.7	9.8	23.3
		5 ppb	-	-	-	-	-	-
		Control	-	-	-	-	-	-
			8 July	7 Aug.	2 Sept.			
2015	Honey	100 ppb	-	115.0	118.0			
		20 ppb	-	19.1	25.5			
		5 ppb	-	4.8	6.4			
		Control	-	-	2.5			
	Adult bees	100 ppb	-	10.6	-			
		20 ppb	-	1.7	-			
		5 ppb	-	-	-			
		Control	-	-	-			
	Wax	100 ppb	-	-	-			
		20 ppb	-	-	-			
		5 ppb	-	-	-			
		Control	-	-	-			
	Syrup	100 ppb	104.0					
		20 ppb	18.7					
		5 ppb	4.6					
		Control	-					

“Year” indicates the year the experiment was initiated. “Conc.” indicates imidacloprid concentration in ppb for that treatment. The limit of detection of imidacloprid was 1 ppb; dashes indicate none detected.

**Fig 2:**
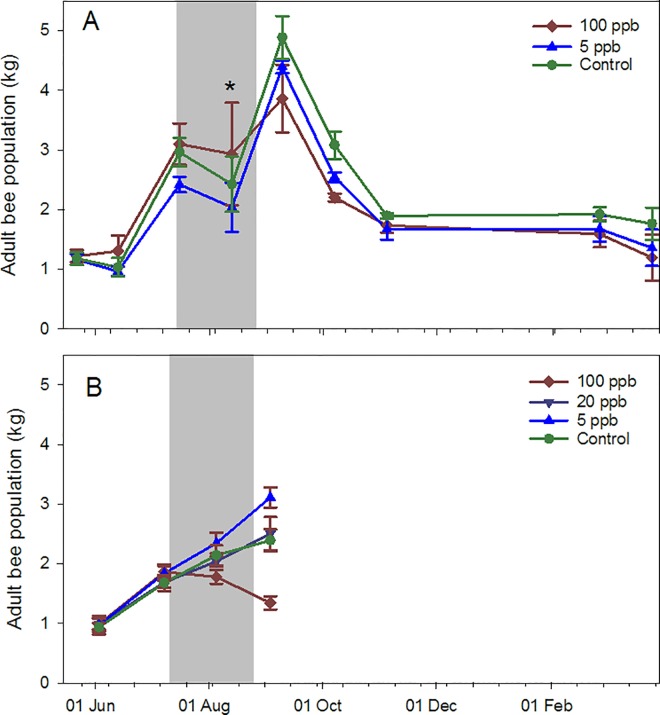
Adult bee populations (kg) for honey bee colonies fed contaminated sugar syrup. The syrup had imidacloprid concentrations of either 100 ppb, 5 ppb or 0 ppb (four colonies per group), in southern Arizona. A) Experiment initiated in 2014; B) Experiment initiated in 2015. Gray zone indicates treatment period. * indicates that masses were estimated using detrended weight amplitudes (see Meikle et al. 2016) rather than inspection data.

**Fig 3:**
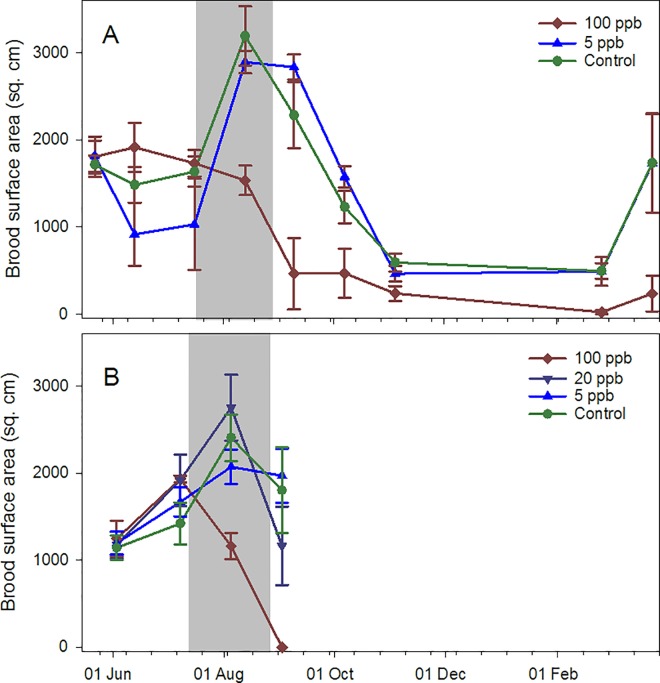
Capped brood surface areas for honey bee colonies fed contaminated sugar syrup. The syrup had imidacloprid concentrations of either 100 ppb, 5 ppb or 0 ppb (four colonies per group), in southern Arizona. A) Experiment initiated in 2014; B) Experiment initiated in 2015. Gray zone indicates time period during which the syrup was given.

Adult bee population (Fig 2), brood area (Fig 3) and average frame weights from the 2014 study were divided into 4 time periods: 1) pre-treatment (22 May to 16 July); 2) during treatment (13 Aug.); 3) post treatment (9 Sept. to 4 Nov.); and 4) post winter (26 Feb. to 23 Mar.) (Table A in S2 File). Pre-treatment no differences were found among groups. During treatment, brood area was significantly lower on average in the 100 ppb group than the 5 ppb and control groups (a weight error prevented adult bee population calculation in Aug.) (see Table 2 and Table B in S2 File). Post treatment, adult bee populations and brood areas were significantly lower among 100 ppb colonies, after controlling for pre-treatment values, than control colonies. Brood area and average frame weight, after controlling for pre-treatment frame weight, were lower in the 100 ppb treatment than the 5 ppb treatment. Post winter, capped brood areas were significantly lower in the 100 ppb group than either the 5 ppb or control groups. Average daily Varroa mite fall over four sampling occasions (Aug., Oct., Nov. and Mar.) were not different across treatments (P = 0.08) but were across sampling occasions (F_3,10_ = 42.82, P<0.0001) with the highest being an average of 19.0 mites per day in Oct. Visual assessment of the colonies revealed no disease symptoms.

**Table 2:** Post hoc comparisons for two field experiments conducted in Arizona.

Year	Time period	Conc.	Response variables					
			Adults	Brood	Frame wt.	Wt. ampl.	Temp. avg.	Temp. ampl.
2014	During treat	100 ppb	-	a	-	-	-	-
		5 ppb	-	**b**	-	-	-	-
		0 ppb	-	**b**	-	-	-	-
	Post treat	100 ppb	a	a	a	a	-	-
		5 ppb	ab	**b**	**b**	**b**	-	-
		0 ppb	**b**	**b**	ab	a	-	-
	Winter	100 ppb	-	a	-	NA	-	**a**
		5 ppb	-	**b**	-	NA	-	b
		0 ppb	-	**b**	-	NA	-	ab
2015	During treat	100 ppb	a	a	NA	-	a	**a**
		20 ppb	**b**	**b**	NA	-	**b**	ab
		5 ppb	**b**	**b**	NA	-	**b**	b
		0 ppb	**b**	**b**	NA	-	**b**	b

“Conc.” indicates imidacloprid concentration in ppb for that treatment. Treatment groups within the same time period with no letters in common are significantly different at α = 0.05 with a Bonferroni comparison for multiple groups. Bold indicates which group or groups had the higher value within each time period and response variable group. Dashes indicate no significant main effect. “NA” indicates comparisons were not conducted.

**Fig 4:**
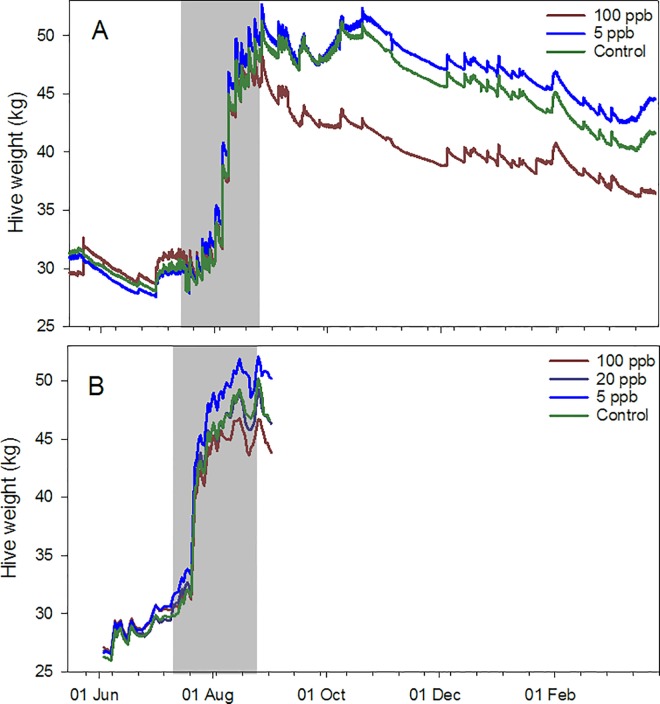
Hourly hive weight over time for honey bee colonies given sugar syrup with imidacloprid. The syrup had imidacloprid concentrations of either 100 ppb, 5 ppb or 0 ppb (four colonies per group), in southern Arizona. A) Experiment initiated in 2014; B) Experiment initiated in 2015. Gray zone indicates treatment period.

**Fig 5:**
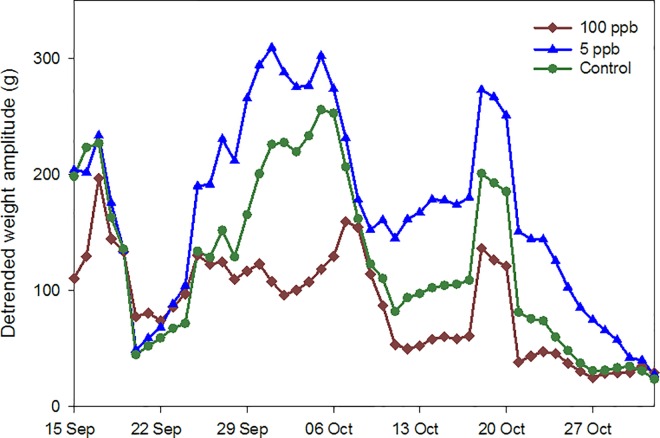
Average amplitudes of sine curves fit to detrended hourly hive weight data. Data were collected during a nectar flow in Sept.-Oct. 2014 from bee colonies given sugar syrup containing imidacloprid concentrations of either 100 ppb, 5 ppb or 0 ppb (four colonies per group), in southern Arizona.

**Fig 6:**
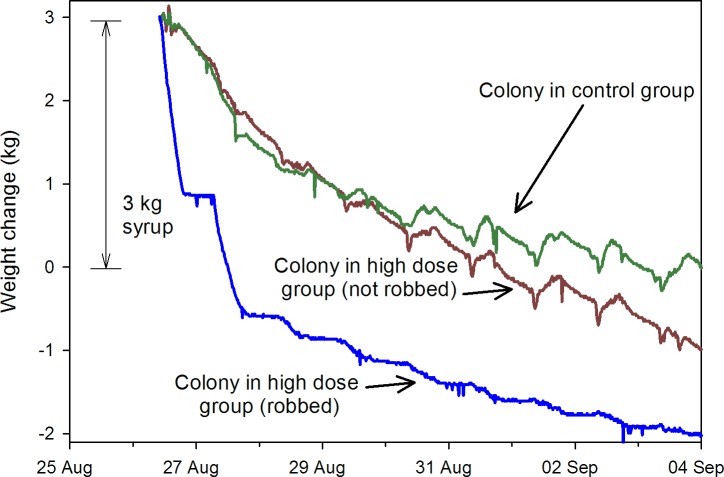
Changes in hourly weight data during a robbing event. Data collected from honey bee colonies involved in a feeding experiment Aug.- Sept. 2014. “High concentration” colonies were fed sugar syrup containing imidacloprid at 100 ppb and “Control” colonies fed sugar syrup alone

Hourly hive weight data from May 2014 to March 2015 showed similar trends (Fig 4). Daily weight amplitudes were divided into three periods: 1) pre-treatment (22 May to 16 July); 2) during treatment (17 July to 26Aug.); and 3) a post treatment nectar flow (3 Sept. to 28 Oct.), with a nectar flow being defined as > 7 d of largely monotonic increases in hive weight (see [21]). No differences were observed among treatments pre- or during treatment (Tables A and B in S3 File), but during a nectar flow starting mid-September, weight amplitudes were significantly higher for colonies in the 5 ppb treatment than for colonies either the 100 ppb or control groups (Fig 5), after controlling for the adult bee population, suggesting greater foraging activity. The data also revealed the robbing of at least one hive in the 100 ppb concentration group. A colony fed syrup is expected to lose weight as moisture in the syrup is driven off by the bees, but a loss rate, even in a desert environment, of > 1.5 kg per day is excessive. A loss rate of 2 kg in < 6 h (Fig 6) indicated robbing.

**Fig 7:**
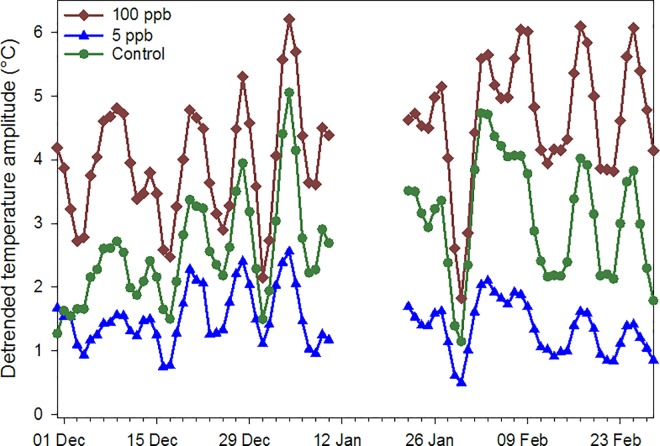
Amplitudes of sine curves fit to detrended hourly hive internal temperature data. Data were collected during winter (Dec. 2014 to Feb. 2015) from honey bee colonies fed sugar syrup containing either 100 ppb, 5 ppb or 0 ppb imidacloprid during July-Aug. 2014.

Temperature data for the 2014 experiment were also divided into three periods: 1) 1) pre-treatment (22 May to 16 July); 2) post treatment (27 Aug. to 30 Nov.); and 3) winter (1 Dec. to 26 Feb.); some data were missing due to equipment problems. Running average temperatures did not differ among treatments during any of those periods (S3 Fig) nor did detrended amplitudes pre- or post-treatment. However, amplitudes in colonies in the 100 ppb treatment group were significantly higher in winter, indicating reduced temperature control, than those in the 5 ppb treatment (Fig 7) but not the control. One hive (100 ppb treatment) died during winter and those data were removed from that analysis.

Discrete data from the 2015 study in Arizona were divided into two time periods, owing to the early termination: 1) pre-treatment (3 June to 8 July); and 2) during treatment (5 Aug. to 2 Sept., which included the final inspection). As in 2014, no significant pre-treatment differences were observed and adult bee populations in the 100 ppb group during treatment were significantly lower than other groups after controlling for pre-treatment values (Tables A and B in S4 File). Similar results were observed for brood surface area. Average frame weights were not affected, but differences may have been obscured by syrup storage from feeding. Average daily Varroa fall data were not different among treatments (P = 0.81).

All syrup was consumed in the 2014 treatment period but not in 2015. Unconsumed syrup was measured on 7 and 24 Aug.; the syrup was replaced with fresh syrup to maintain similar imidacloprid exposure between the two experiments. Colonies in the 100 ppb treatment left on average 4.21 kg of syrup while colonies in the other groups left 0.50–0.80 kg. These data were analyzed with similar data from Arkansas (see below).

**Fig 8:**
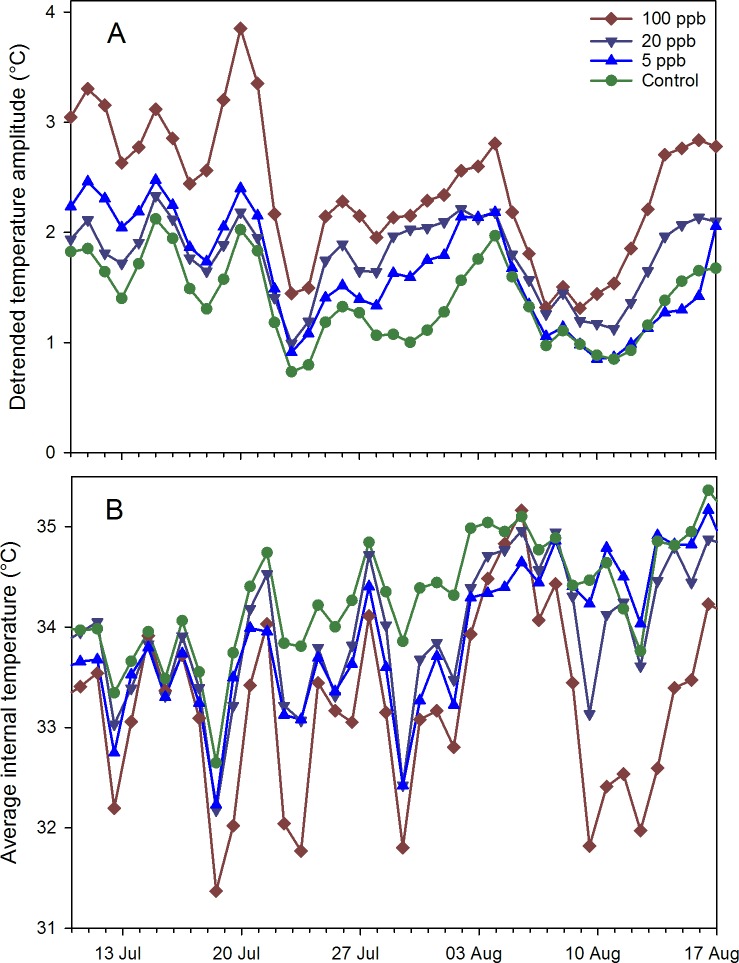
Internal temperature data for honey bee colonies fed contaminated sugar syrup. The syrup contained either 100 ppb, 20 ppb, 5 ppb or 0 ppb imidacloprid during July-Aug. 2015. A) Amplitudes of sine curves fit to detrended hourly internal temperature data; B) Average daily hive temperature.

Continuous weight and temperature data in 2015 were grouped into the same pre-treatment and during treatment time periods as the hive inspection data. Weight amplitudes were not different among treatments, but the lack of a nectar flow would have made any differences more difficult to detect. Average temperatures, with adult bee population as a covariate, were not different pre-treatment but were significantly lower for the 100 ppb treatment than for the 20 ppb, 5 ppb and control groups during treatment. Temperature amplitudes in the 100 ppb treatment were again significantly higher than either the 5 ppb treatment or control but not the 20 ppb treatment (Fig 8).

### Field experiment-Arkansas

Neonicotinoid residue analysis differed in two important respects from the Arizona studies: 1) imidacloprid levels were lower in Arkansas post treatment, particularly in honey (Table 3) than in the Arizona studies, and were also lower in the syrup itself for the 100 ppb treatment; and 2) measurable amounts of another neonicotinoid pesticide, clothianidin, were detected with imidacloprid in some samples of bees, wax and nectar, always at concentrations of <7 ppb.

**Table 3:** Imidacloprid concentrations, in ppb, in adult bees, honey, pollen and wax after exposure of bee colonies to imidacloprid in sugar syrup in Arkansas.

Material	Treatment group	Aug. 31	Sept. 29
Adult bees	100 ppb	6.8±1.0	3.7±1.5
	20 ppb	-	-
	5 ppb	-	-
	Control	-	-
Honey	100 ppb	23.8±10.3	28.6±3.5
	20 ppb	5.3±0.7	7.2±0.8
	5 ppb	1.5±0.9	0.6±0.6
	Control	-	-
Pollen	100 ppb	1.9±0.6	0.5 ±0.5
	20 ppb	0.6±0.5	-
	5 ppb	0.5±0.5	-
	Control	-	-
Wax	100 ppb	1.6±0.5	-
	20 ppb	-	-
	5 ppb	-	-
	Control	-	-
Syrup	100 ppb	66.4	
	20 ppb	17.3	
	5 ppb	8.1	
	Control	0	

The limit of detection of imidacloprid was 1 ppb; dashes indicate none detected.

No differences among groups with respect to brood surface area, average frame weight or total hive weight were detected, after controlling for pre-existing differences, after the start of treatment. The exposure of colonies to clothianidin may have affected differences among treatment groups. Unconsumed syrup was measured on 24 July, 20 and 28 Aug. Colonies in the 100 ppb treatment left on average over 5.42 kg of syrup while colonies in the other treatment groups left on average 1.05 to 1.74 kg. Treatment group had a significant effect on the log amount of consumed syrup (F_3,27_ = 7.92, P = 0.0017) but site did not and, as noted above, no pre-existing size differences among groups were detected. Colonies in the 100 ppb group consumed less syrup than those in other groups (P = 0.0042, 0.0013 and 0.0047 for the 20 ppb, 5 ppb and control groups, respectively).

### Field experiment-Mississippi

Coumaphos residues were detected in honey and adult bees sampled before treatment (Table 4). Both coumaphos and imidacloprid were detected in honey and adult bees post treatment. Imidacloprid levels were consistent with those observed in Arizona; it was also detected in honey sampled from colonies that had not been fed imidacloprid, indicating robbing, so comparisons between the 0 and 5 ppb imidacloprid treatments should be interpreted cautiously.

**Table 4:** Coumaphos and imidacloprid concentrations, in ppb, detected in honey and adult bees before start of treatment (16 July) and after 32 days (17 Aug.) in Mississippi with respect to treatment group.

Material	Coumaphos conc.	Imidacloprid conc.	16 July		17 Aug.	
			Coumaphos	Imidacloprid	Coumaphos	Imidacloprid
Honey	5.8 ppm	20 ppb	Trace	-	54.2	23.6
	5.8 ppm	5 ppb	-	-	202	6.9
	5.8 ppm	None	-	-	60	4.4
	None	100 ppb	-	-	Trace	86.3
	None	20 ppb	-	-	-	19
	None	5 ppb	-	-	-	11.7
	None	None	-	-	-	2.3
Adult	5.8 ppm	20 ppb	Trace	-	260	5.1
bees	5.8 ppm	5 ppb	11.3	-	104	2.5
	5.8 ppm	None	-	-	111	3.3
	None	100 ppb	-	-	-	18.9
	None	20 ppb	-	-	-	4.6
	None	5 ppb	-	-	-	2.7
	None	None	-	-	-	-

The limit of detection of imidacloprid was 1 ppb and that of coumaphos was 5 ppb; “Trace” indicates positive coumaphos detection but <5 ppb; dashes indicate none detected.

**Fig 9:**
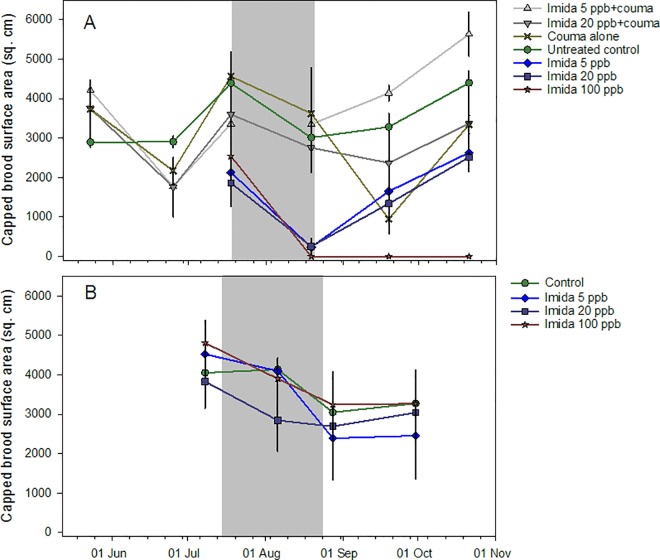
Capped brood levels among honey bee colonies at two experimental sites. Colonies were fed either 100, 20, 5 or 0 ppb imidacloprid in sugar syrup over approximately 6 weeks at two locations. A) Mississippi apiary, which also included some hives given coumaphos at 5.8 ppm; B) Arkansas apiary. Gray area indicates treatment period.

Exposure to coumaphos in patties did not have a significant effect for the May, June or July datasets on capped brood area (P = 0.35) or on the number of frame spaces (P = 0.22, 0.66 and 0.10, respectively, using nonparametric ANOVA). Analysis of all groups after syrup feeding showed a significant effect of imidacloprid, but not coumaphos, and a significant interaction of imidacloprid and coumaphos (Tables A and B in S5 File.) (Fig 9). Only one post hoc contrast was significant, showing that colonies not given coumaphos but fed 20 ppb imidacloprid syrup had significantly less brood than those given both coumaphos and 5 ppb imidacloprid. Excluding colonies fed coumaphos, control colonies had more capped brood on average than colonies in other groups and colonies fed 5 ppb had more brood than those fed 100 ppb (S4 Table). *Varroa* densities, measured as mites per bee, were not different across treatments (P = 0.51) or sampling occasions (P = 0.052); densities reached 1.9 mites per bee in Oct.

## Discussion

Sublethal concentrations of neonicotinoid pesticides can affect several aspects of honey bee behavior and ecology. The focus of this work was on colony-level behaviors such as brood rearing, foraging activity and temperature management over time, across different environments. Cage studies in controlled conditions were conducted to focus on adult bee survivorship and consumption. No differences were found in survivorship. Bees in cages fed 100 ppb imidacloprid consumed less syrup on average than those in other groups but the differences were not large. Interestingly, caged bees in the 2015 study consumed less syrup on average, 18.5 mg per bee per day, than those in the 2014 study, 22.6 mg; the reason for this difference is not known. Caged bees ate on average 420 mg (100 ppb group) to 480 mg (control group) sugar from emergence to 50 d. Sugar consumption by an adult honey bee has been estimated to be 534 to 1005 mg (pollen and nectar foragers, respectively) from emergence to 31 d [19]. The lower consumption rate of syrup by caged bees would result in a lower imidacloprid exposure compared to actively-foraging field bees. This was born out in residue analyses. Caged bees were restricted to treatment syrup but imidacloprid was detected only once in two studies (at <2 ppb in the 100 ppb treatment) while bees from that treatment group in Arizona field studies had 6.8 to 19.3 ppb imidacloprid. Imidacloprid is metabolized within hours by bees [1] so those concentrations probably represent mostly undigested syrup in the gut.

Determination of colony-level effects of sublethal exposure is difficult, largely due to natural variation among colonies and to uncontrolled factors such as differential exposure of experimental colonies to exogenous agrochemicals, and up to 24 to 80 colonies per treatment have been suggested [25]. We observed differences with far fewer colonies per treatment although not at all sites. In Arizona, colonies in the 100 ppb imidacloprid treatment group had significantly less brood than other groups during and post treatment. Colonies in the 2014 experiment also had fewer adult bees and lighter frames post treatment, indicating less stored food. Temperature variability, as measured by daily temperature amplitudes, were higher on average in the 100 ppb group than those in the 5 ppb group during winter for the 2014 study and during treatment for the 2015 study. Temperature data can be affected by the distance of the bee cluster to the sensor, which can vary among colonies and over time [26]; we assumed that distance was random with respect to treatment. Low temperature variability has been associated with brood rearing and high variability with little or no brood [21].

High coumaphos concentrations, such as 100 ppm or greater, affect queens by reducing oviposition and ovarian weight, causing early supercedure and queen cell rejection [27], and increasing worker mortality [22]. In the Mississippi study presented here, coumaphos levels were lower (5.8 ppm) and coumaphos alone did not have a significant impact on the amount of brood, although its interaction with imidacloprid was significant. Among colonies not fed coumaphos, imidacloprid exposure had a negative effect on brood production at all concentrations. In interpreting these results, the effects of unknown local conditions cannot be ruled out for two reasons: 1) differences in capped brood area in coumaphos-free groups fed syrup with 5 and 0 ppb imidacloprid were not observed at any other sites; and 2) imidacloprid was detected in honey from both the coumaphos positive and coumaphos negative control groups by the end of treatment, indicating robbing. No significant differences in brood or frame weights were observed in Arkansas. Experimental execution and environment between Arkansas and the other sites differed in several respects: 1) Arkansas colonies were not deprived of stored honey; 2) syrup in the 100 ppb treatment in Arkansas apparently had less imidacloprid than planned; and 3) Arkansas colonies were exposed to other agrochemicals, including another neonicotinoid, clothianidin.

Syrup consumption differed among treatment groups, sites and between years. Colonies in the 100 ppb treatment groups in Arizona in 2015 (but not 2014) and in Arkansas left more syrup than colonies in other groups, on average by a factor of at least three. Colonies in Arizona had few alternatives for nectar; the lack of consumption may have been due to lower activity levels (reduced foraging activity was observed) or to a dislike for the treated syrup [9]. Reduced consumption was observed in the 100 ppb group in the cage studies, and newly-emerged honey bees can detect imidacloprid at < 3 ppb [28]. Colonies in Arkansas may have sought other nectar sources. Some colonies in Mississippi may have avoided high imidacloprid exposure by absconding: hives exposed to 0, 5, and 20 ppb retained their original queens but three of four hives fed 100 ppb syrup absconded by September, one of which was recovered later with the original queen and overwintered successfully in new equipment. Interestingly, consumption rates were also different across years for caged bees, which would not have had the option of alternative forage or absconding.

Colonies in the 5 ppb and control groups had more brood area, heavier frames and greater temperature control than colonies in the 100 ppb groups. In addition, colonies in the 5 ppb group had higher daily weight amplitudes, which is associated with flight activity, during a nectar flow post treatment (in 2014) than colonies in other groups, including the control. Average frame weights, which would reflect nectar storage, were not different between the 5 ppb and control groups, so either the flight activity did not increase foraging success, or consumption by the colonies was higher. Imidacloprid is a nicotine analog and affects insect nicotinic acetylcholine receptors [29]. Honey bees have been found to have a tolerance for the “trace” concentrations of nicotine, 0.1 to 5 ppm, found naturally in some nectars [30] and the survival of bees from “weak” colonies (colonies exhibiting a lack of vigor for unknown reasons) improved when placed in cages and given a 300 μM solution of nicotine, or about 48.6 ppm [31]. That concentration is several magnitudes higher than a 5 ppb imidacloprid solution.

These experiments involved a particular kind of exposure to imidacloprid (via syrup) with exposure to constant concentrations over 5–6 weeks. Honey bees can be exposed to pesticides via other means, including in pollen, seed treatment dusts and water and means of exposure may play a role in the impact on colony health. Exposing bees via contaminated supplemental pollen feed (e.g., [2, 32]) may impact nurse bees and larvae more than it would foragers because those life stages feed more on pollen than foragers [33, 34]. While an effort was made to harmonize colony management among sites in this study, colonies had site-specific pesticide exposures, due to agriculture, pre-existing contaminants in drawn comb, and bee pest treatments that should be taken into account when interpreting the data across sites. Regarding the treatments selected here, even in agriculturally intensive areas, such as the southeastern U.S., concentrations as high as 100 ppb have seldom been reported in pollen or nectar. Stewart et al. [35] found that neonicotinoid levels in soils and wild flowers after seed treatment applications in experimental and commercial plots seldom exceeded 50 ppb. Bees exposed to high levels of imidacloprid in nectar also may simply reduce or avoid feeding on contaminated nectar, whether or not alternative forage is available [9]. Detection of effects of low concentrations of a neonicotinoid pesticide on colony level behavior suggests that the interaction between pollinators and commercial agriculture is more complex than previously thought.

### Conclusions

- Imidacloprid concentrations were stable in stored nectar or honey in the hive environment for at least 7 months;
- Bee colonies with little stored honey and fed an imidacloprid concentration of 100 ppb in syrup generally had lower adult bee populations, less capped brood and worse winter thermoregulation compared to controls;
- Syrup consumption differed with respect to imidacloprid concentration as well as year in cage studies, and with respect to imidacloprid concentration, year and site in field studies; caged bees or bee colonies fed 100 ppb syrup often consumed less than other groups;
- Within-day hive weight changes were significantly higher, indicating increased foraging activity, in the 5 ppb treatment than either the 100 ppb treatment or the control during a nectar flow.

## Supporting Information

S1 Fig Diagram of treatments for Mississippi trial. (PDF)

S2 Fig Total water and syrup consumption for bees kept in cages and fed sugar syrup with imidacloprid concentrations of either 100 ppb, 20 ppb, 5 ppb or 0 ppb. (PDF)

S3 Fig Average daily internal temperatures for bee colonies subjected to one of three treatments: fed syrup containing 100 ppb imidacloprid, fed syrup containing 5 ppb imidacloprid, and fed syrup with no imidacloprid. (PDF)

S1 File **Figs. A-D**. Intercept and scale parameters, and associated distribution scale and shape parameters, for a regression-based fit of a Weibull distribution with censoring to bee survivorship in cage studies over two trials.
(PDF)

S2 File **TablesA and B**. Analysis and post hoc contrast results for hive inspection data from a field experiment conducted in Arizona 2014.
(PDF)

S3 File **Tables A and B**. Analysis and post hoc contrast results for continuous weight and temperature data from a field experiment conducted in Arizona 2014.
(PDF)

S4 File **Tables A and B.** Analysis and post hoc contrast results for hive inspection and continuous monitoring data from a field experiment conducted in Arizona 2015.
(PDF)

S5 File **Tables A and B.** Analysis and post hoc contrast results from a field experiment conducted in Mississippi 2015.
(PDF)

S6 File Experimental data. (XLSX)

S1 Table Summary of experimental designs. (PDF)

S2 Table Preparation of pesticide treatments. (PDF)

S3 Table Analysis of select parameters from Weibull distributions to cage survivorship data. (PDF)

S4 Table Analysis and post hoc contrast results showing the effect of exposure to imidacloprid on capped brood area for hives not exposed to coumaphos from a field experiment conducted in Mississippi 2015. (PDF)
