# Supplementary figures and images for: Sublethal Effects of Imidacloprid on Honey Bee Colony Growth and Activity at Three Sites in the U.S
Source: PLoS One. 2016 Dec 28;11(12):e0168603. doi: 10.1371/journal.pone.0168603 (PMC5193417; doi:10.1371/journal.pone.0168603)

**S1 Fig.** Diagram of treatments for Mississippi trial.

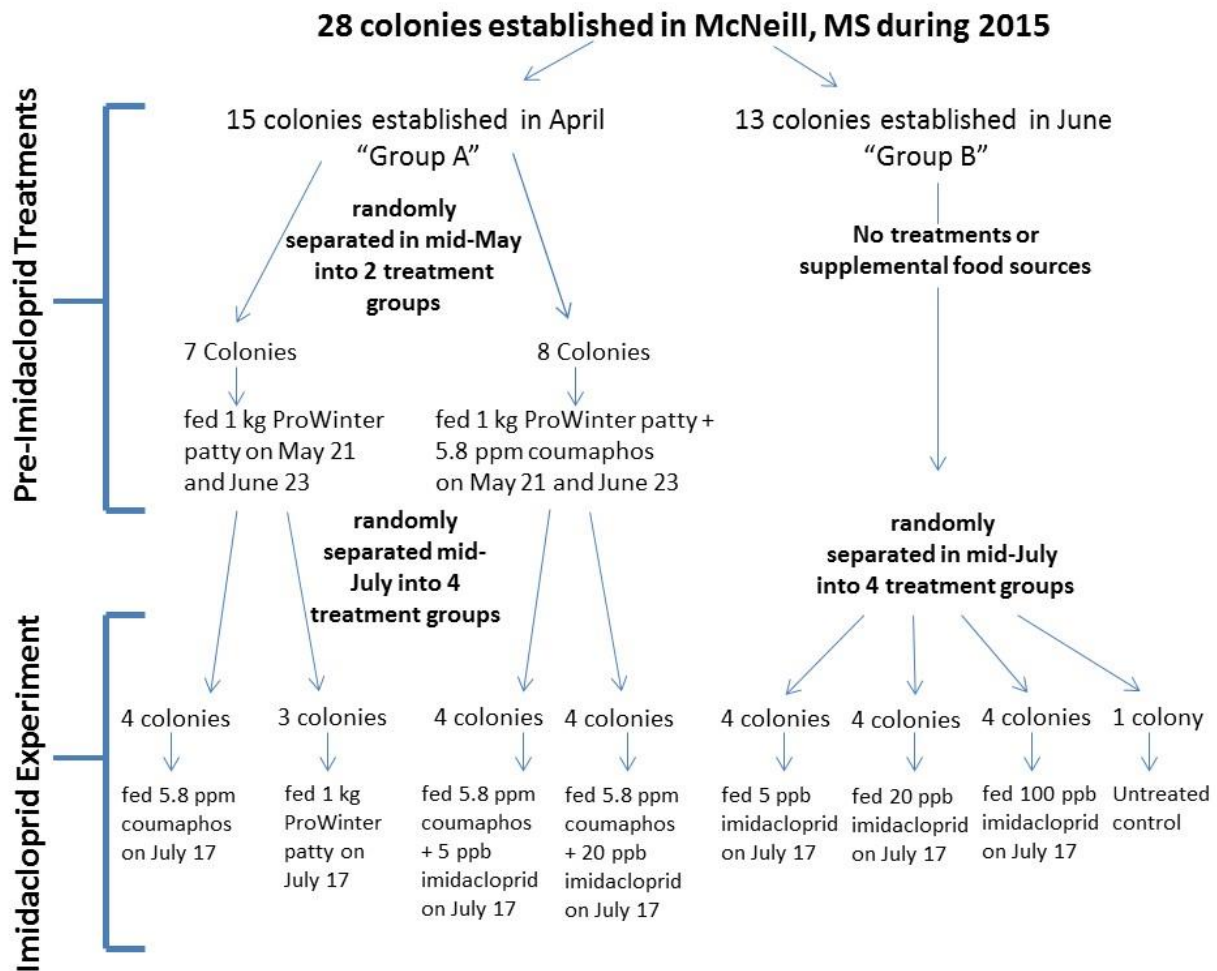

Supplement: S1 Fig — (PDF) [file pone.0168603.s001.pdf]
