# Supplementary material for: Sublethal Effects of Imidacloprid on Honey Bee Colony Growth and Activity at Three Sites in the U.S
Source: PLoS One. 2016 Dec 28;11(12):e0168603. doi: 10.1371/journal.pone.0168603 (PMC5193417; doi:10.1371/journal.pone.0168603)

S6 Fig. Total water and syrup consumption for bees kept in cages and fed sugar syrup with imidacloprid concentrations of either 100 ppb, 20 ppb, 5 ppb or 0 ppb. Each cage was stocked with 100 bees (7-8 cages per group). “First experiment” was conducted Sept-Oct. 2014 (no 20 ppb treatment) and “second experiment” was conducted Aug.-Sept. 2015

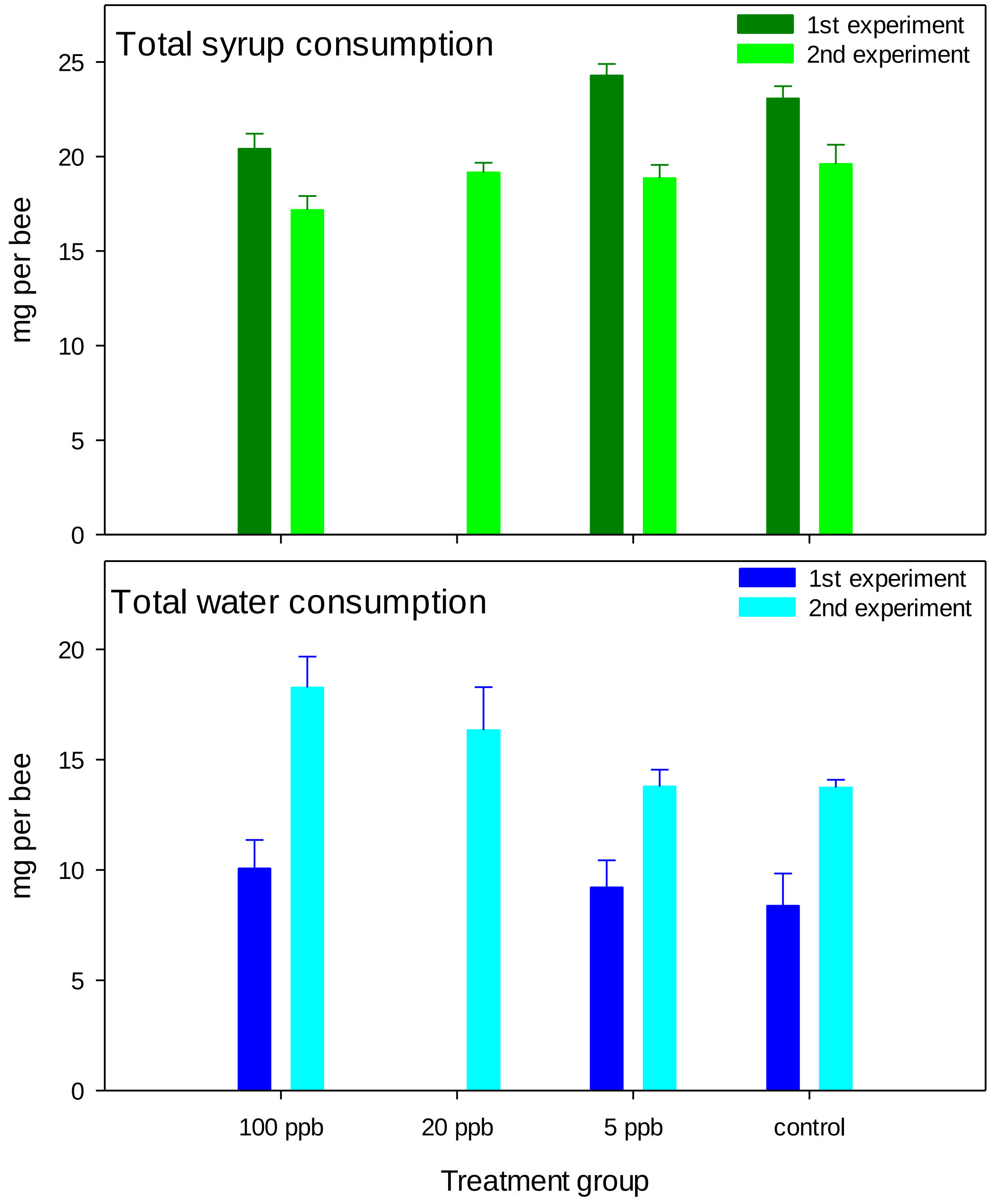

Supplement: S2 Fig — (PDF) [file pone.0168603.s002.pdf]
