# Supplementary material for: Sublethal Effects of Imidacloprid on Honey Bee Colony Growth and Activity at Three Sites in the U.S
Source: PLoS One. 2016 Dec 28;11(12):e0168603. doi: 10.1371/journal.pone.0168603 (PMC5193417; doi:10.1371/journal.pone.0168603)

S3 Fig. Average daily internal temperatures for bee colonies subjected to one of three treatments: fed syrup containing 100 ppb imidacloprid, fed syrup containing 5 ppb imidacloprid, and fed syrup with no imidacloprid. Black line shows ambient temperature obtained from a local weather station.

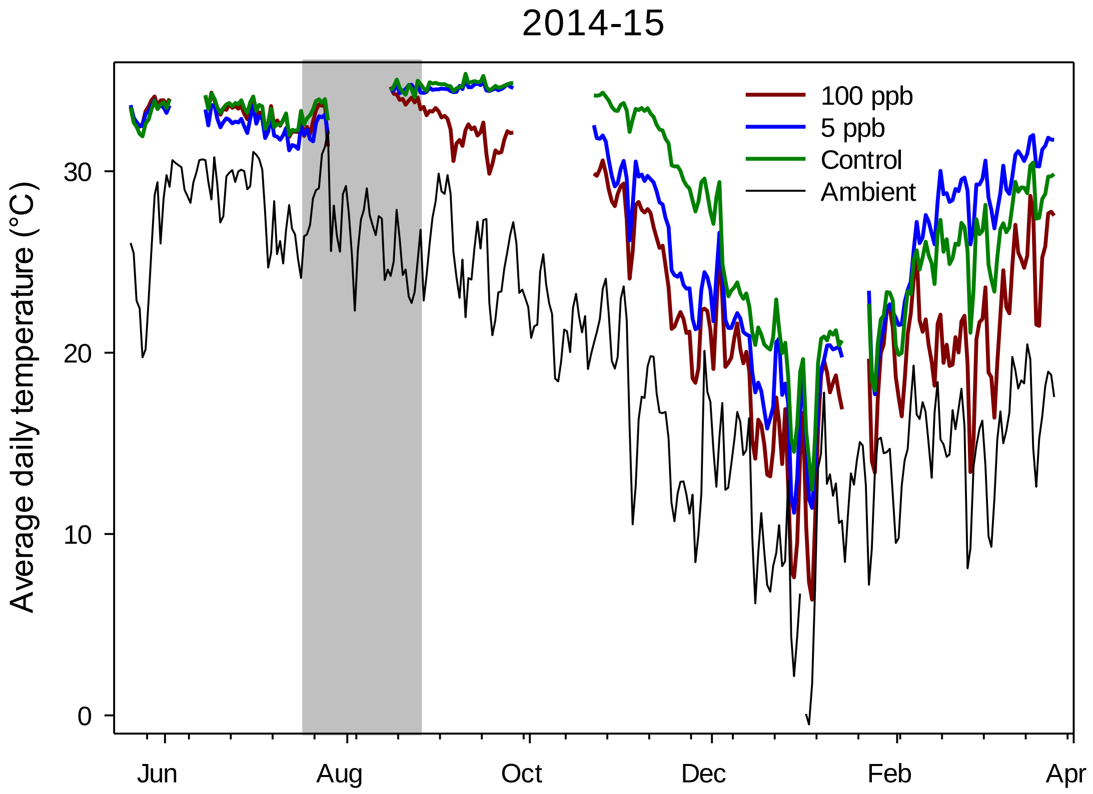

Supplement: S3 Fig — (PDF) [file pone.0168603.s003.pdf]
