# Supplementary material for: Sublethal Effects of Imidacloprid on Honey Bee Colony Growth and Activity at Three Sites in the U.S
Source: PLoS One. 2016 Dec 28;11(12):e0168603. doi: 10.1371/journal.pone.0168603 (PMC5193417; doi:10.1371/journal.pone.0168603)

Fig. A. Intercept parameter for a regression-based fit of a Weibull distribution with censoring to bee survivorship in cage studies over two trials. Lines show confidence limits (α=0.05).

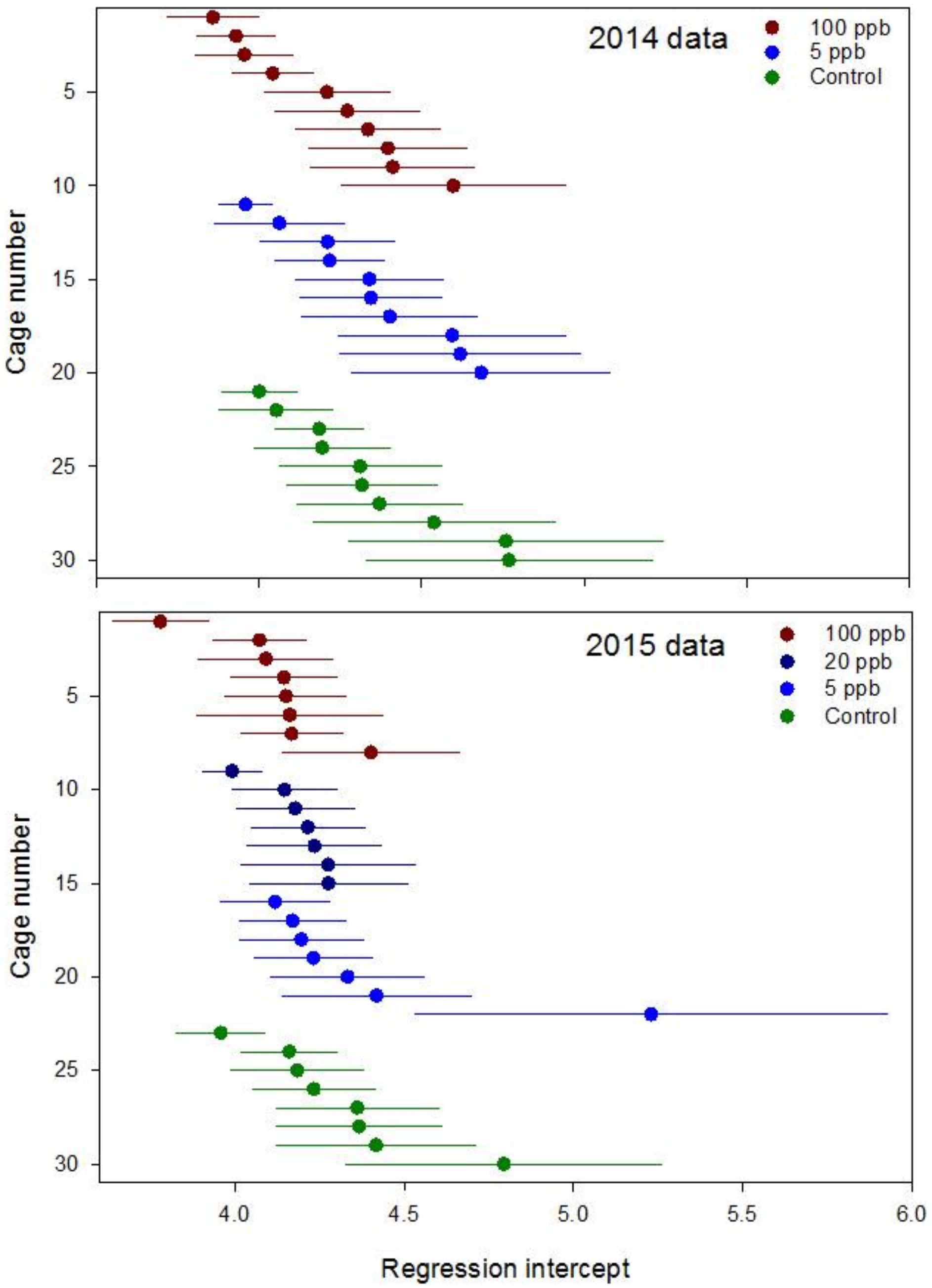

Fig. B. Scale parameter for a regression-based fit of a Weibull distribution with censoring to bee survivorship in cage studies over two trials. Lines show confidence limits (α=0.05).

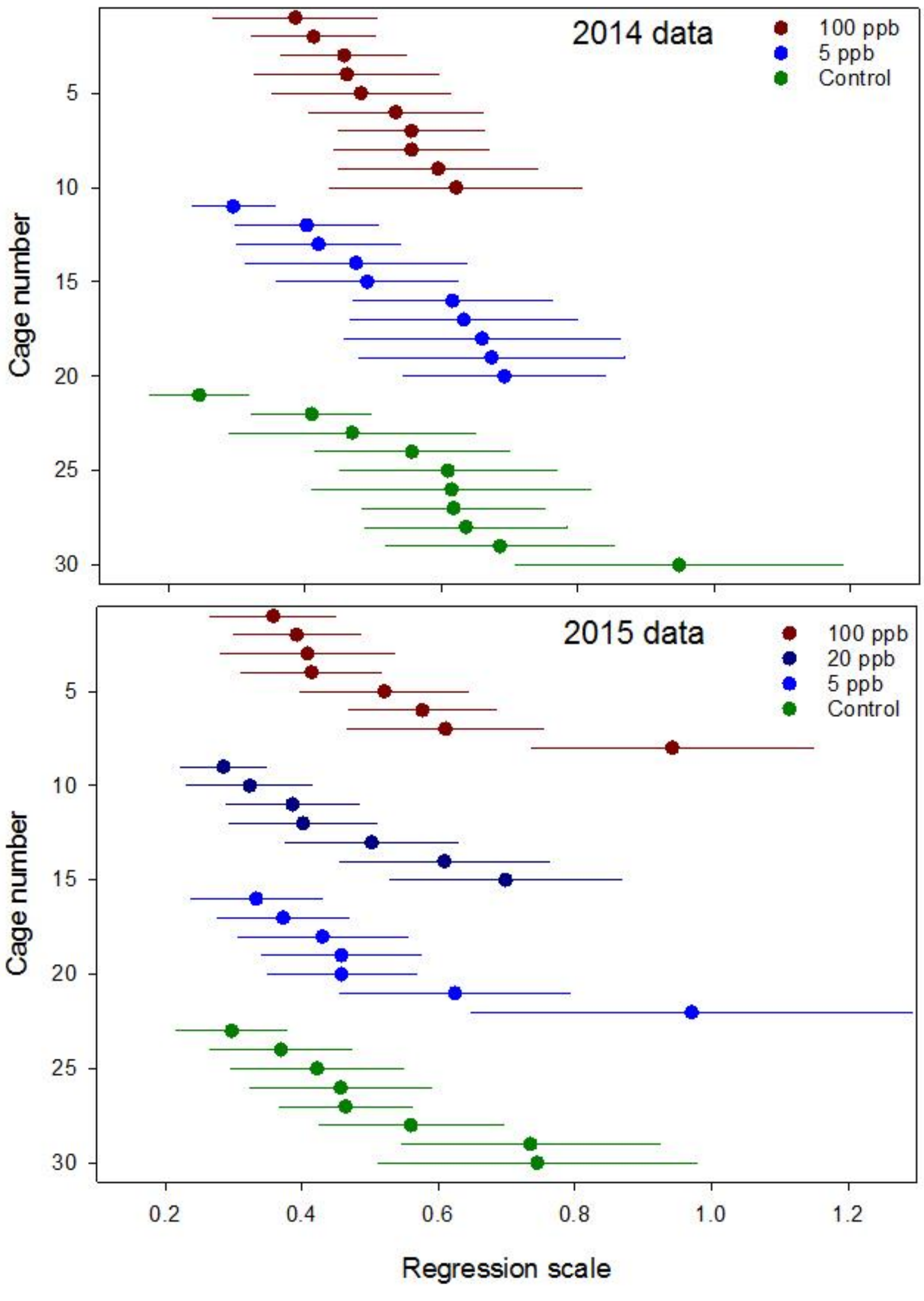

Fig. C. Scale parameter, in d, for Weibull distributions with censoring fit to bee survivorship in cage studies over two trials. Lines show confidence limits (α=0.05).Note that the confidence limits for one cage of the 5 ppb treatment in the 2015 dataset are only partially shown due to its size.

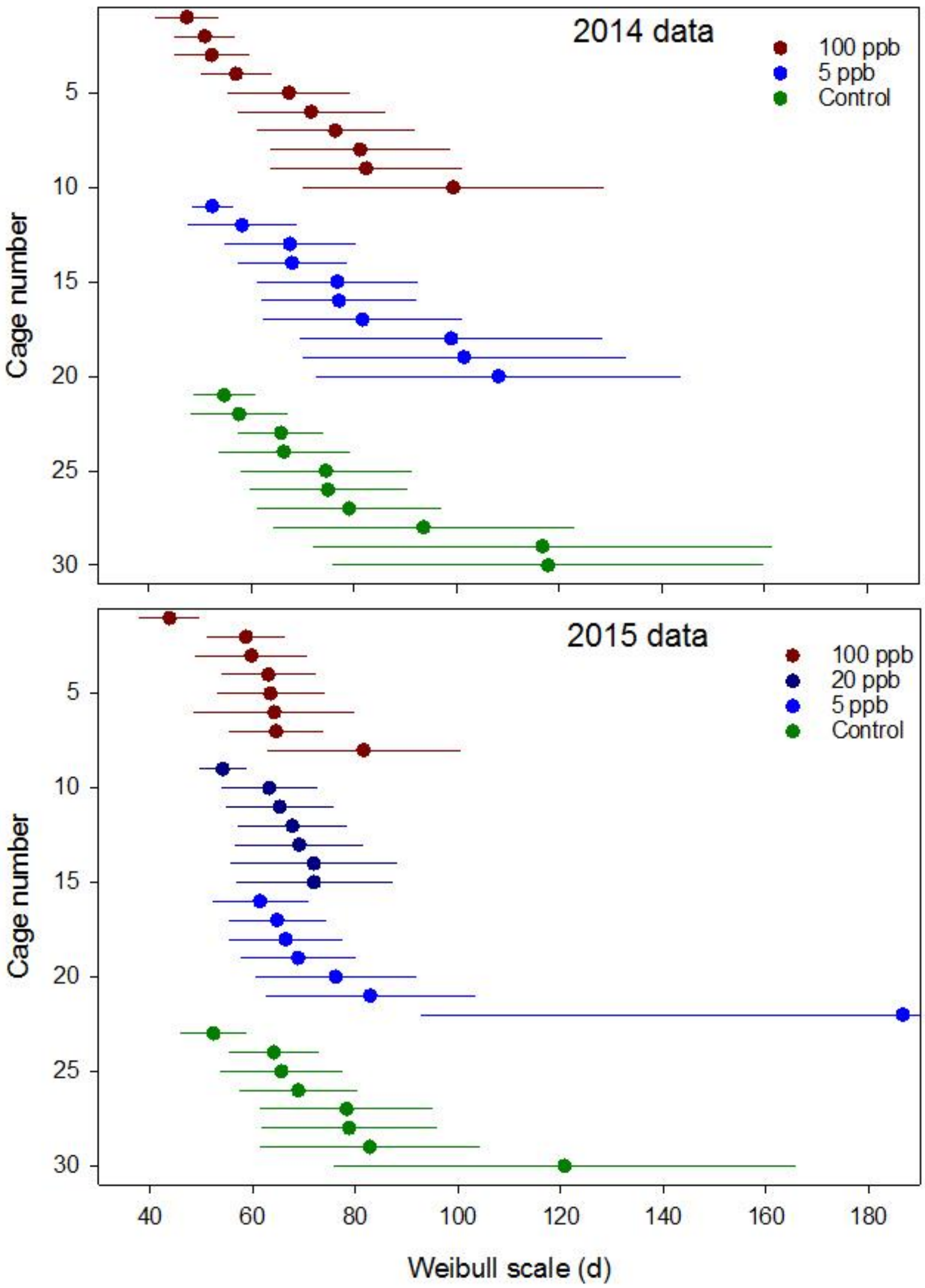

Fig. D. Shape parameter for Weibull distributions with censoring fit to bee survivorship in cage studies over two trials. Lines show confidence limits (α=0.05)

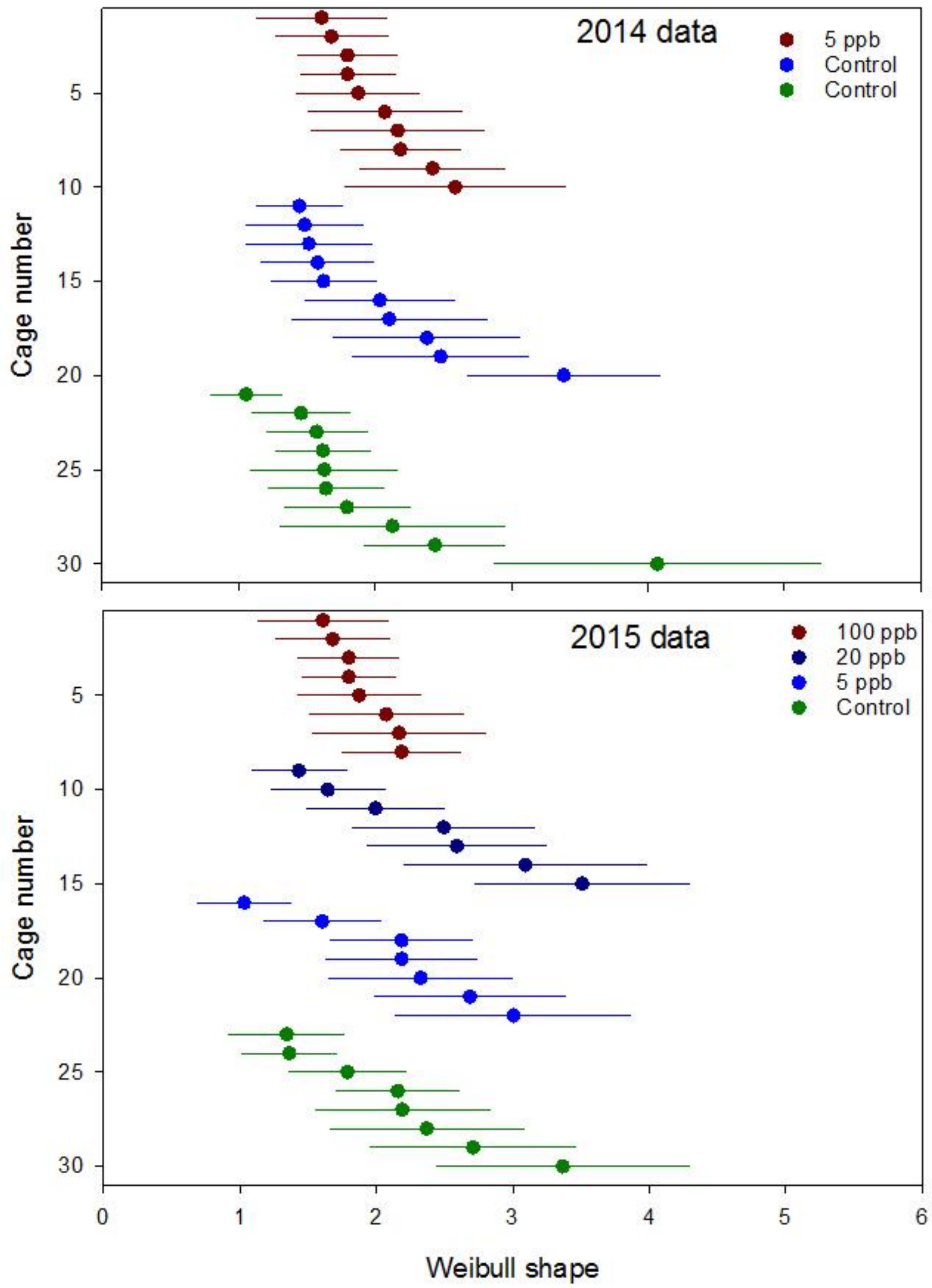

Supplement: S1 File — Figs. A-D. Intercept and scale parameters, and associated distribution scale and shape parameters, for a regression-based fit of a Weibull distribution with censoring to bee survivorship in cage studies over two trials. (PDF) [file pone.0168603.s004.pdf]
