# Supplementary material for: Sublethal Effects of Imidacloprid on Honey Bee Colony Growth and Activity at Three Sites in the U.S
Source: PLoS One. 2016 Dec 28;11(12):e0168603. doi: 10.1371/journal.pone.0168603 (PMC5193417; doi:10.1371/journal.pone.0168603)
Supplement: S2 File — TablesA and B. Analysis and post hoc contrast results for hive inspection data from a field experiment conducted in Arizona 2014. (PDF) [file pone.0168603.s005.pdf]

Table A. Analysis of hive inspection data from a field experiment conducted in Arizona 2014. "Transf." refers to the data transformation to improve normality, "Brood area" refers to the surface area of capped brood and "Adult bee population" refers to the total mass of adult bees in kg. "NS" indicates that the "Imidacloprid" factor was not significant at α=0.05; the main effects for those analyses are shown. Analyses were conducted using mixed-model ANOVA, with adult bee population estimates prior to treatment used as covariates. For each analysis, an appropriate covariance structure was chosen using the Akaike Information Criterion. Degrees of freedom were calculated using the Kenward-Roger method.

Period	Dep. var.	Transf.	Covar. struct.	Factors	Num. d.f.	Den. d.f.	F	P
During treat	Brood area	none	-	Imidacloprid	2	9	14.41	0.0016
	Frame wt.			Imid., Date, Pre-treat frame wt.				NS
Post treat	Adult bee population	none	ante(1)	Imidacloprid	2	21.9	4.65	0.0028
				Date	2	15.5	260.87	<0.0001
				Imid.*Date	4	17.5	2.85	0.0709
				Pre-treat adult bee population	1	26	1.93	0.0203
	Brood area	none	un	Imidacloprid	2	27	14.96	<0.0001
				Date	2	27	23.58	<0.0001
				Imid.*Date	4	27	5.22	0.0030
	Frame wt.	none	un	Imidacloprid	2	26	5.45	0.0106
				Date	2	8.0	3.70	0.0730
				Imid.*Date	4	8.9	2.88	0.0873
				Pre-treat frame wt.	1	26	4.13	0.0524
Post winter	Adult bee population			Imid., Date, Pre-treat ad. bee population				NS
	Brood area	Sq. rt.	un	Imidacloprid	2	8	6.86	0.0184
				Date	1	8	26.57	0.0009
				Imid.*Date	2	8	1.17	0.3591
	Frame wt.			Imid., Date, Pre-treat frame wt.				NS

Table B. Post hoc contrast results for imidacloprid exposure on hive inspection data from a field experiment conducted in Arizona 2014. "Value" refers to the value of the contrast. Dashes indicate there was no significant main effect.

Time period	Contrast	Adult bee population		Brood		Frame wt.	
		Value	P	Value	P	Value	P
During treat	100 vs 5 ppb	-	-	-1359	0.0077	-	-
	100 vs 0 ppb	-	-	-1658	0.0021	-	-
	5 vs 0 ppb	-	-	-299	1.000	-	-
Post treat	100 vs 5 ppb	-553	0.063	-945	<0.0001	-409	0.0175
	100 vs 0 ppb	-745	0.0024	-749	0.0015	-342	0.0560
	5 vs 0 ppb	-193	1.000	196	0.93	67	1.0000
Winter	100 vs 5 ppb	-	-	-20.6	0.0349	-	-
	100 vs 0 ppb	-	-	-20.9	0.0321	-	-
	5 vs 0 ppb	-	-	-0.4	1.000	-	-
