# Supplementary material for: Sublethal Effects of Imidacloprid on Honey Bee Colony Growth and Activity at Three Sites in the U.S
Source: PLoS One. 2016 Dec 28;11(12):e0168603. doi: 10.1371/journal.pone.0168603 (PMC5193417; doi:10.1371/journal.pone.0168603)
Supplement: S3 File — Tables A and B. Analysis and post hoc contrast results for continuous weight and temperature data from a field experiment conducted in Arizona 2014. (PDF) [file pone.0168603.s006.pdf]

Table A. Statistical results for continuous weight and temperature data from a field experiment conducted in Arizona 2014. "Transf." refers to the data transformation to improve normality, "Brood area" refers to the surface area of capped brood and "Adult bee population" refers to the total mass of adult bees in kg. "NS" indicates that the "Imidacloprid" factor was not significant at α=0.05; the main effects for those analyses are provided. Analyses were conducted using mixed-model ANOVA, with adult bee population estimates prior to treatment used as a covariate. For each analysis, an appropriate covariance structure was chosen using the Akaike Information Criterion. Degrees of freedom were calculated using the Kenward-Roger method.

Period	Dep. var.	Transf.	Covar. struct.	Factors	Num. d.f.	Den. d.f.	F	P
Post treat	Weight ampl.	log	ante(1)	Imidacloprid	2	25.61	16.83	<0.0001
				Date	11	16.12	33.27	<0.0001
				Imid.*Date	22	20.42	1.39	0.2285
				Adult bee population	1	26.83	20.23	0.0001
Post treat	Temp. ampl.			Imid., Date				NS
	Temp. avg.			Imid., Date, Adult bee population				NS
Winter	Temp. ampl.	log	ante(1)	Imidacloprid	2	10.13	5.92	0.0198
				Date	15	18.41	8.47	<0.0001
				Imid.*Date	30	22.76	0.66	0.8600
				Adult bee population	1	13.96	0.27	0.6131
	Temp. avg			Imid., Date, Adult bee population				NS

Table B. Post hoc contrast results for imidacloprid exposure on continuous weight and temperature data from a field experiment conducted in Arizona 2014. "Value" refers to the value of the contrast. Dashes indicate there was no significant main effect. "NA" indicates the analysis was not conducted; weight amplitudes are a function of foraging traffic and honey bees generally do not forage in winter.

Period	Contrast	Hive weight amplitudes		Temperature amplitudes	
		Value	P	Value	P
Post treat	100 vs 5 ppb	-0.216	<0.0001	-	-
	100 vs 0 ppb	-0.027	1.000	-	-
	5 vs 0 ppb	0.189	0.0004	-	-
Winter	100 vs 5 ppb	NA		0.485	0.0186
	100 vs 0 ppb	NA		0.2381	0.3801
	5 vs 0 ppb	NA		-0.2464	0.3400
