# Supplementary material for: Sublethal Effects of Imidacloprid on Honey Bee Colony Growth and Activity at Three Sites in the U.S
Source: PLoS One. 2016 Dec 28;11(12):e0168603. doi: 10.1371/journal.pone.0168603 (PMC5193417; doi:10.1371/journal.pone.0168603)
Supplement: S4 File — Tables A and B. Analysis and post hoc contrast results for hive inspection and continuous monitoring data from a field experiment conducted in Arizona 2015. (PDF) [file pone.0168603.s007.pdf]

Table A. Analysis of hive inspection and continuous monitoring data from a field experiment conducted in Arizona 2015. "Transf." refers to the data transformation to improve normality, "Brood area" refers to the surface area of capped brood and "Adult bee population" refers to the total mass of adult bees in kg. "NS" indicates that the "Imidacloprid" factor was not significant at α=0.05; the main effects for those analyses are provided. Analyses were conducted using mixed-model ANOVA, with adult bee population estimates prior to treatment used as covariates. For each analysis, an appropriate covariance structure was chosen using the Akaike Information Criterion. Degrees of freedom were calculated using the Kenward-Roger method.

Period	Dep. var.	Transf.	Covar. struct.	Factors	Num. d.f.	Den. d.f.	F	P
During treat	Adult bee population	none	ante(1)	Imidacloprid	3	18	17.25	<0.0001
				Date	1	18	5.14	0.0360
				Imid.*Date	3	18	5.77	0.0060
				Pre-treat adult bee population	1	18	7.83	0.0119
	Brood area	none	ar(1)	Imidacloprid	3	11.7	6.91	0.0062
				Date	1	7.9	48.91	0.0001
				Imid.*Date	3	7.9	8.06	0.0088
				Pre-treat adult bee population	1	11.9	0.25	0.6275
	Temp. ampl.	log	ante(1)	Imidacloprid	3	19.4	6.04	0.0044
				Date	10	25.3	17.53	<0.0001
				Imid.*Date	30	32.1	1.77	0.0574
	Temp. avg	square	ante(1)	Imidacloprid	3	31.5	15.59	<0.0001
				Date	10	40.2	21.42	<0.0001
				Imid.*Date	30	75.7	3.02	<0.0001
				Adult bee population	1	29.4	0.91	0.3473
	Weight ampl.			Imid., Date, Adult bee population				NS

Table B. Post hoc contrast results for imidacloprid exposure on hive inspection and continuous monitoring data from a field experiment conducted in Arizona 2015. "Value" refers to the value of the contrast.

Contrast	Adult bee population		Brood		Temperature amplitude		Average temperature	
	Value	P	Value	P	Value	P	Value	P
100 vs 20 ppb	-912	0.0012	-1028	0.0317	0.138	0.135	-57.7	0.0004
100 vs 5 ppb	-1231	<0.0001	-1052	0.0188	0.169	0.0389	-58.4	0.0046
100 vs 0 ppb	-939	0.0024	-1230	0.0119	0.229	0.0038	-87.0	<0.0001
20 vs 5 ppb	-320	0.671	-24	1.000	0.032	1.000	-0.7	1.000
20 vs 0 ppb	-27	1.000	-201	1.000	0.092	0.747	-29.4	0.1178
5 vs 0 ppb	293	1.000	-178	1.000	0.060	1.000	-28.7	0.1995
