# Supplementary material for: Sublethal Effects of Imidacloprid on Honey Bee Colony Growth and Activity at Three Sites in the U.S
Source: PLoS One. 2016 Dec 28;11(12):e0168603. doi: 10.1371/journal.pone.0168603 (PMC5193417; doi:10.1371/journal.pone.0168603)
Supplement: S5 File — Tables A and B. Analysis and post hoc contrast results from a field experiment conducted in Mississippi 2015. (PDF) [file pone.0168603.s008.pdf]

Table A. Analysis of a field experiment conducted in Mississippi 2015. “Transf.” refers to the data transformation to improve normality, and “Brood area” refers to the surface area of capped brood. “NS” indicates that the “Imidacloprid” factor was not significant at α=0.05; the main effects for those analyses are provided. Analyses were conducted using mixed-model ANOVA, with adult bee population estimates prior to treatment used as covariates. For each analysis, an appropriate covariance structure was chosen using the Akaike Information Criterion. Degrees of freedom were calculated using the Kenward-Roger method.

Period	Dep. var.	Transf.	Covar. struct.	Factors	Num. d.f.	Den. d.f.	F	P
Apr.-July	Brood area			Coumaphos, Date				NS
Aug.-Nov.	Brood area	none	ante(1)	Coumaphos	1	19.34	3.11	0.0934
				Imidacloprid	2	18.23	3.7	0.0449
				Couma*Imid	2	17.64	8.72	0.0023
				Date	2	53	42.47	<0.0001
				Date*Couma	2	53	4.29	0.0188
				Date *Imid	4	53	3.07	0.024
				Date*Couma*Imid	4	53	0.64	0.636
				Occupied spaces	1	18.9	0.72	0.4073

Table B. Post hoc contrast results (1st group – 2nd group) for the effects of imidacloprid exposure on capped brood data from a field experiment conducted in Mississippi 2015. "Couma" refers to the presence or absence of added coumaphos to the bee diet in the spring, "Imida" refers to the concentration of imidacloprid in ppb in sugar syrup fed to bees in July and August. "Value" refers to the value of the contrast.

1st group		2nd group		Capped brood area	
Couma	Imida	Couma	Imida	Value	P
no	0	no	5	69.85	0.584
no	0	no	20	91.37	0.052
no	0	yes	0	47.46	1.000
no	0	yes	5	-31.97	1.000
no	0	yes	20	34.14	1.000
no	5	yes	0	-22.39	1.000
no	5	yes	5	-101.82	0.090
no	5	yes	20	-35.71	1.000
no	20	no	5	-21.52	1.000
no	20	yes	0	-43.91	1.000
no	20	yes	5	-123.34	0.005
no	20	yes	20	-57.23	0.962
yes	0	yes	5	-79.44	0.114
yes	0	yes	20	-13.33	1.000
yes	20	yes	5	-66.11	0.255
