# Supplementary material for: Sublethal Effects of Imidacloprid on Honey Bee Colony Growth and Activity at Three Sites in the U.S
Source: PLoS One. 2016 Dec 28;11(12):e0168603. doi: 10.1371/journal.pone.0168603 (PMC5193417; doi:10.1371/journal.pone.0168603)
Supplement: S1 Table — (PDF) [file pone.0168603.s010.pdf]

S1 Table. Summary of experimental designs.

Site	Experiment dates	Treatment dates	Treatment groups	Total no. replicates	Sampling occasions	Response variables	Residue analyses
AZ	9 Sept.-31 Oct. 2014	16 Sept.-31 Oct.	0, 5 and 100 ppb imidacloprid	30 cages	Daily mortality; weekly consumption	Survivorship; syrup and water consumption	Adult bees
AZ	27 July-16 Sept. 2015	3 Aug.-16 Sept.	0, 5, 20 and 100 ppb imidacloprid	30 cages	Same as above	Same as above	Same as above
AZ	May 2014-Mar. 2015	17 July-26 Aug.	0, 5 and 100 ppb imidacloprid	12 colonies	Monthly except Dec. and Jan.	Adult bee population; capped brood area; frame weight; continuous hive weight amplitudes; temperature average and amplitudes; Varroa fall	Adult bees; wax; honey
AZ	May–Aug. 2015	9 July-25 Aug.	0, 5, 20 and 100 ppb imidacloprid	16 colonies	Monthly	Same as above	Same as above
AR	May-Nov. 2015	Same as above	Same as above	Same as above	Same as above	Capped brood area; frame weight; total hive weight	Adult bees; wax; honey; pollen
MS-part 1	Apr.-Oct. 2015	21 May-21 June	0 and 5.8 ppm coumaphos	15 colonies	Same as above	Capped brood area; frame spaces; Varroa density	Adult bees; honey
MS-part 2	June-Oct. 2015	17 July-19 Aug.	0, 5, 20 and 100 ppb imidacloprid and 5.8 ppm coumaphos	Colonies from MS- part 1 plus 13 colonies	Same as above	Same as above	Adult bees; honey
