# Supplementary material for: Sublethal Effects of Imidacloprid on Honey Bee Colony Growth and Activity at Three Sites in the U.S
Source: PLoS One. 2016 Dec 28;11(12):e0168603. doi: 10.1371/journal.pone.0168603 (PMC5193417; doi:10.1371/journal.pone.0168603)
Supplement: S2 Table — (PDF) [file pone.0168603.s011.pdf]

S2 Table. Preparation of pesticide treatments.

Treatment material	Procedure
Imidacloprid syrup treatment preparation.	Treatment solutions were made fresh the day of each feeding using analytical-grade imidacloprid (Pestanal, CAS # 138261-41-3). Formulation of 1 kg of treatment solution were applied using a 1 L Nalgene bottle (Thermo Scientific, Rochester, NY) as follows:<br>1. Control (0 ppb imidacloprid) sucrose solution were mixed at 1:1 w:w (e.g. 500 g sucrose:500mL distilled water). Sucrose was dissolved in distilled water using a large mixing flask with mixing bar on a hot plate heated to no more than 60°C.<br>2. Sucrose solution for solutions with imidacloprid was mixed as above but 100 mL was withheld (thus “short”) to allow for the added volume of respective imidacloprid spikes. 500 g of sugar is dissolved in 400 mL of distilled water to allow for the addition of a 100 mL spike to achieve 1 kg of treatment solution. 900 g of “short” sugar solution was transferred to a Nalgene bottle, then the spike added to each individual bottle.<br>3. A 10 ppm imidacloprid stock solution was made by dissolving 1.0 mg of imidacloprid, in 100 mL of distilled water, using a mixing bar but without heat. To avoid problems with static electricity, the imidacloprid was weighed into a small, nonreactive plastic receptacles and those receptacles were placed in the solution, the solution stirred, and the receptacles removed after confirming the imidacloprid had dissolved.<br>4. For the 5 ppb solution: 0.5 mL of the stock solution was mixed into 99.5 mL of distilled water to achieve 100 mL of spike solution, which was then added to 900g of the short sucrose solution to achieve 1 kg of 5 ppb imidacloprid syrup. For the 20 ppb solution (only in 2nd experiment) 2.0 mL of stock solution was mixed into 98.0 mL of distilled water, and that solution added to 900 g of the short solution to achieve 1 kg of 20 ppb imidacloprid syrup. For the 100 ppb solution: 10.0 mL of stock solution was mixed into 90.0 mL of distilled water, and that solution was then added to 900g of the short sucrose solution to achieve 1 kg of 100 ppb imidacloprid syrup.
Coumaphos patty preparation.	Coumaphos was formulated as follows: 46.4 mg analytical-grade coumaphos (Pestanal, CAS # 56-72-4) was dissolved in 5 ml acetone (CAS # 67-64-1; Sigma-Aldrich, MO). This solution was thoroughly mixed directly into 1 kg Pro Winter carbohydrate patty (Mann Lake Ltd., Hackensack, MN), which in turn was thoroughly mixed into 3 kg of patty, and that amount mixed into 8 kg patty.
Coumaphos syrup preparation	Coumaphos sugar syrup was prepared by dissolving 116 mg of coumaphos in 10 ml of acetone and then adding that solution to 20 kg of a 1:1 (w/w) aqueous sugar solution. A control treatment was prepared by mixing only acetone in 20 kg sugar syrup. Both solutions were stored at 4 ^{0}C until use.
