# Supplementary material for: Sublethal Effects of Imidacloprid on Honey Bee Colony Growth and Activity at Three Sites in the U.S
Source: PLoS One. 2016 Dec 28;11(12):e0168603. doi: 10.1371/journal.pone.0168603 (PMC5193417; doi:10.1371/journal.pone.0168603)
Supplement: S3 Table — (PDF) [file pone.0168603.s012.pdf]

**S3 Table.** Analysis of select parameters from Weibull distributions to cage survivorship data. Denominator degrees of freedom were 47 for the first analysis and 26 for the second.

| Datasets | Factors | Num. d.f. | 30$^{th}$ percentile | | 50$^{th}$ percentile | | 30$^{th}$-40$^{th}$ percentiles | |
|---|---|---|---|---|---|---|---|---|
| | | | F | P | F | P | F | P |
| 2014+2015 | Treatment | 2 | 2.49 | 0.0940 | 2.80 | 0.0707 | 0.85 | 0.4381 |
| | Year | 1 | 0.01 | 0.9318 | 0.02 | 0.8876 | 0.11 | 0.7388 |
| | Treat. x Year | 2 | 0.39 | 0.6788 | 0.41 | 0.6641 | 0.30 | 0.7463 |
| 2015 | Treatment | 3 | 2.25 | 0.1058 | 1.83 | 0.1665 | 0.93 | 0.4365 |
