# Supplementary material for: Sublethal Effects of Imidacloprid on Honey Bee Colony Growth and Activity at Three Sites in the U.S
Source: PLoS One. 2016 Dec 28;11(12):e0168603. doi: 10.1371/journal.pone.0168603 (PMC5193417; doi:10.1371/journal.pone.0168603)
Supplement: S4 Table — (PDF) [file pone.0168603.s013.pdf]

**S4 Table**. Analysis and post hoc contrast results results showing the effect of exposure to imidacloprid on capped brood area for hives not exposed to coumaphos from a field experiment conducted in Mississippi 2015. Analyses were conducted using mixed-model ANOVA, with adult bee population estimates prior to treatment used as covariates. The ante(1) covariance structure was chosen using the Akaike Information Criterion. Degrees of freedom were calculated using the Kenward-Roger method. Post hoc contrasts, using the Bonferroni correction, are shown at the bottom.

| Effect | Num DF | Den DF | F Value | Adj P |
|---|---|---|---|---|
| Imidacloprid | 3 | 11.51 | 23.19 | <0.0001 |
| Sampling occasion | 2 | 11.22 | 20.82 | 0.0002 |
| Imida x sampl. occ. | 6 | 12.86 | 3.02 | 0.0455 |
| Occupied spaces | 1 | 10.93 | 0.52 | 0.4846 |

| Post hoc contrasts | DF | Estimate | Standard Error | Adj P |
|---|---|---|---|---|
| 0 ppb vs 5 ppb | 12.8 | 78.9 | 18.7 | 0.0077 |
| 0 ppb vs 20 ppb | 11.6 | 96.4 | 16.9 | 0.0007 |
| 0 ppb vs 100 ppb | 12.2 | 143.1 | 17.7 | <0.0001 |
| 20 ppb vs 5 ppb | 11.4 | -17.5 | 16.6 | 1.0000 |
| 100 ppb vs 5 ppb | 11.0 | -64.2 | 16.1 | 0.0119 |
| 100 ppb vs 20 ppb | 11.0 | -46.7 | 16.1 | 0.0842 |
